# Author Correction: Enhancing energetic disorder in all-organic composite dielectrics for high-temperature capacitive energy storage

**DOI:** 10.1038/s41467-025-62435-0

**Published:** 2025-07-31

**Authors:** Tan Zeng, Li Meng, Qiao Li, Dongduan Liu, Qian Zhou, Jinliang He, Qi Li, Chao Yuan

**Affiliations:** 1 College of Electrical and Information Engineering, Hunan University, Changsha, Hunan China; 2 State Key Laboratory of Power System, Department of Electrical Engineering, Tsinghua University, Beijing, China

**Keywords:** Electronic devices, Electronic materials, Energy storage

Correction to: *Nature Communications* 10.1038/s41467-025-60741-1, published online 1 July 2025

In the version of the article initially published, the names of the authors appeared in the order “surname, given name” and have now been amended to “given name, surname” in the HTML and PDF versions of the article.

