# Supplementary material for: Enhancing energetic disorder in all-organic composite dielectrics for high-temperature capacitive energy storage
Source: Nat Commun. 2025 Jul 1;16:5620. doi: 10.1038/s41467-025-60741-1 (PMC12216148; doi:10.1038/s41467-025-60741-1)
Supplement: Supplementary file 1 — Supplementary Information [file 41467_2025_60741_MOESM1_ESM.pdf]

Supplementary Information

Enhancing Energetic Disorder in All-organic Composite Dielectrics for High-temperature Capacitive Energy Storage

Tan Zeng [1,&], *Li Meng* [2,&], *Qiao Li*[*1], *Dongduan Liu* [1], *Qian Zhou* [1], *Jinliang He*[2], *Qi Li*[*2], *Chao Yuan*[*1]

[1]*College of Electrical and Information Engineering, Hunan University, Changsha, Hunan 410082, China*

[2]*State Key Laboratory of Power System, Department of Electrical Engineering, Tsinghua University, Beijing 100084, China*

[&]These authors contributed equally: Tan Zeng, Li Meng

Author to whom correspondence should be addressed: chaoyyy@outlook.com; qili1020@tsinghua.edu.cn; qiaoli@hnu.edu.cn;

Table of Contents

Supplementary Note 1 4
Supplementary Note 2 4
Supplementary Note 3 5
Supplementary Note 4 5
Supplementary Note 5 5
Supplementary Note 6 6
Supplementary Note 7 6
Supplementary Note 8 7
Supplementary Note 9 8
Supplementary Note 10 10
Supplementary Note 11 12
Supplementary Note 12 13
Supplementary Note 13 14
Supplementary Note 14 15
Supplementary Note 15 15
Supplementary Figure S1 16
Supplementary Figure S2 16
Supplementary Figure S3 17
Supplementary Figure S4 17
Supplementary Figure S5 18
Supplementary Figure S6 18
Supplementary Figure S7 19
Supplementary Figure S8 20
Supplementary Figure S9 20
Supplementary Figure S10 21
Supplementary Figure S11 21
Supplementary Figure S12 22
Supplementary Figure S13 22
Supplementary Figure S14 23
Supplementary Figure S15 24
Supplementary Figure S16 25
Supplementary Figure S17 25
Supplementary Figure S18 26

Supplementary Figure S19 26
Supplementary Figure S20 27
Supplementary Figure S21 27
Supplementary Figure S22 28
Supplementary Figure S23 29
Supplementary Figure S24 29
Supplementary Figure S25 30
Supplementary Figure S26 31
Supplementary Figure S27 32
Supplementary Figure S28 33
Supplementary Figure S29 34
Supplementary Table S1 35
Supplementary Figure S30 36
Supplementary Table S2 37
Supplementary Figure S31 38
Supplementary Table S3 39
Supplementary Figure S32 40
Supplementary Figure S33 41
Supplementary Figure S34 42
Supplementary Figure S35 42
Supplementary Figure S36 43
Supplementary Figure S37 44
Supplementary Figure S38 45
Supplementary Figure S39 46
Supplementary Figure S40 47
Supplementary Figure S41 48
Supplementary Figure S42 49
Supplementary Figure S43 50
Supplementary Figure S44 51
Supplementary Figure S45 52
Supplementary Table S4 53
Supplementary Table S5 55
Supplementary References 57

Supplementary Note 1

Sample Preparation for fs-TAS and TRPL mapping

The preparation of samples for both fs-TAS and TRPL mapping involves a series of critical steps, meticulously designed to ensure the precision and reproducibility of the measurements. Initially, a 2 cm × 2 cm quartz glass substrate was selected for its outstanding optical transparency and stability, making it an optimal base for subsequent film deposition and optical analysis. To prepare the poly(amic acid) (PAA) solution, a specified mass of BPADA and MDA was dissolved in 10 mL of NMP, and the mixture was stirred at room temperature for 12 hours. Concurrently, high-polarity organic molecules, such as DADQ, DMABN, and PTCNQ, were dissolved in NMP at a concentration of 5 mg/mL and sonicated for 3 hours. The solution containing these organic molecules was then combined with the PAA solution in the desired proportions, resulting in a composite solution. This composite solution was used to cast a thin film onto the quartz glass substrate via spin-coating. The film thickness was carefully controlled by adjusting both the solution concentration and spin-coating parameters. Specifically, the composite solution was spin-coated at 3000 rpm on a heated spin platform (set to 80 °C), yielding a film with an approximate thickness of 200 nm. Finally, the film was dried in a vacuum oven at 200 °C for 12 hours. This rigorous sample preparation protocol ensures that the films possess high optical quality and surface uniformity, essential for obtaining reliable results in both fs-TAS and TRPL mapping experiments.

Supplementary Note 2

Nano-IR Measurement Procedures:

For Nano-IR analysis, the samples were carefully prepared by cutting the films into uniform square sections (typically 1 cm×1 cm) and adhering them onto metal substrates, ensuring minimal stress to prevent deformation. The measurements were conducted using an Anasys nanoIR3 system (Bruker), equipped with a quantum cascade laser (QCL) covering the spectral range of 900-3600 cm^{-1} in tapping AFM-IR mode. The samples were irradiated with a pulsed, tunable infrared source (optical parametric oscillator), emitting 10 ns pulses at a repetition rate of 1 kHz, with a beam spot size of approximately 30 μm. The induced oscillations of the AFM probe, recorded as the AFM-IR signal, were subsequently analyzed through fast Fourier transform to extract the corresponding spectral data.

Supplementary Note 3

KPFM measurements Procedures:

The PAA solution, incorporating high-polarity small molecules, was prepared prior to spin-coating. The resulting mixture was then sequentially spin-coated onto gold-coated substrates with low roughness to ensure uniform electrostatic measurements. After thorough cleaning and drying, the samples were subjected to atomic force microscopy (AFM) to capture surface morphology images using a Bruker Dimension Icon AFM in tapping mode. Kelvin probe force microscopy (KPFM) measurements were conducted in dual-pass mode to minimize tip-sample interactions and obtain accurate surface potential mapping. The scan rate was set to 0.5 Hz to achieve high-resolution surface potential maps with a spatial resolution between 20 and 50 nm. To prevent contamination or electrical interference, all measurements were performed in a controlled environment, maintaining consistent humidity and temperature.

Supplementary Note 4

Measurements of the nano-isothermal surface potential decay

Initially, a single line in the composite was selected as a reference baseline for the charging and ISPD measurements. Charges were injected by applying a DC voltage to the probe tip and repetitively scanning for approximately 5 minutes to ensure adequate charge injection into the measured film. Subsequently, after removing the DC voltage, KPFM measurements were performed at multiple time intervals to observe changes in surface potential over time. This approach provided a time-resolved view of charge dissipation dynamics at various positions along the scanned line.

Supplementary Note 5

Measurements of the steady-state photoluminescence (PL) microscopy

To evaluate the impact of energetic disorder within the material, steady-state PL microscopy measurements were employed, providing a sensitive probe for detecting spatial disorder in the composite films. This approach involves the continuous excitation of the sample using a light source of appropriate energy and power, with the excitation either focused onto a micrometer-sized spot using confocal optics or uniformly applied over a larger area. The emitted PL signal is collected and directed

into a spectrograph, where the dispersed light is captured by a highly sensitive detector. The detected optical signal is then converted into an electrical signal by electronic components, allowing for visualization and analysis of local variations in emission intensity across the sample surface.

Supplementary Note 6

Measurements of the femtosecond transient absorption spectroscopy (fs-TAS)

Femtosecond transient absorption spectroscopy (fs-TAS) is a powerful technique for monitoring electron transfer kinetics in polymers. In this method, the laser system generates a femtosecond laser pulse centered at 390 nm, which is split into two beams of different intensities via a beam splitter. The stronger beam passes through a chopper and then through the optical parametric amplifier (OPA) to produce a short-wavelength pump pulse, which excites the sample from the ground state to an excited state. Simultaneously, the less intense beam passes through an optical delay line and interacts with a sapphire crystal, generating a continuous white light pulse that acts as the probe pulse. At the sample, both the pump and probe pulses overlap spatially, while their temporal overlap is regulated by the optical delay line, which adjusts the path difference to introduce a controlled time delay. The probe pulse that traverses the sample is detected, whereas the pump pulse is blocked by a stopper. Differential optical absorption spectra are obtained by measuring the probe pulse's absorption with and without pump irradiation. By systematically varying the delay time between the pump and probe pulses, the changes in probe signal intensity are monitored, indicating fluctuations in the population of charge carriers in the excited state. Through this approach, the relaxation kinetics of charge carriers transitioning from the excited to the ground state can be elucidated.

Supplementary Note 7

Measurements of the time-resolved photoluminescence mapping

We employed a home-built photoluminescence photoluminescence-scanned imaging microscope, integrated with a time-correlated single photon counting (TCSPC)

module, to map the PL kinetics within the polymer film at elevated temperature. The film sample was excited using a pulse laser (PIXEA-CU-1, AUREA, France) with a wavelength of 375 nm, a repetition rate of 5 MHz, and a pulse width of ~35 ps. The excitation laser beam was focused on the sample through a 100× air objective lens (NA = 0.95, Olympus SLMPlan N, 100×), achieving a spot radius of 1.02 μm ($1/e^2$ of the maximum intensity measured with an EMCCD camera, DU-897U-CS0-#BV, Andor, UK). By positioning the excitation laser spot at a specific location on the film or cross-section of the device, rapid scanning via a galvanometer mirror allowed for efficient photon collection. Each scanning image consisted of 256 × 256 pixels. The fluorescence signal was captured using a high-speed detector (HPM-100-50, Hamamatsu, Japan) equipped with a 660 nm long-pass filter and a 680 nm long-pass filter.

Supplementary Note 8

Simulation of charge diffusion of polymer films

The Gaussian Disorder Model (GDM) can thus be expressed in the following form:

$$\mu(F,T)=\mu_{\infty}\exp\left[-\left(\frac{2\sigma}{3k_BT}\right)^2\right]\cdot\exp\left(\beta\sqrt{F}\right) \tag{S1}$$

Where σ represents the energetic disorder, characterizing the width of the Gaussian distribution of the density of energy states for the carrier transport sites. μ_{∞} denotes the mobility at infinite temperature. k is the Boltzmann constant. T is the temperature, and F is the electric field. According to the GDM, increasing the energetic disorder will result in a substantial decline in charge mobility.

The second governing equation describes the current density J_{bul}, which quantifies the movement of charge carriers under an applied electric field. In the presence of energetic disorder, the current density is determined by the mobility, charge carrier density ρ, and the applied electric field (E). Mathematically, this relationship is expressed as:

$$J_{bul}=-\mu(E,\sigma)\cdot\rho\cdot E \tag{S2}$$

where μ (E, σ) represents the mobility, which is temperature-and disorder-dependent as described previously, ρ is the charge carrier density, indicating the number of charge carriers per unit volume, and E is the applied electric field driving the charge carriers

through the polymer matrix.

The third equation, the continuity equation, governs charge conservation within the material, ensuring that any temporal changes in charge density (ρ) align with the current density and the presence of any sources or sinks of charge. This equation is given by:

$$\frac{\partial \rho}{\partial t} + \nabla \cdot J_{bul} = 0 \tag{S3}$$

where ρ denotes the local charge density, and the term $\nabla \cdot J_{bul}$ represents the divergence of the current density, which accounts for the spatial variation in charge density.

By solving these coupled equations using COMSOL Multiphysics, we can systematically model charge transport behavior in disordered polymer composites. This comprehensive simulation framework enables us to investigate the fundamental mechanisms governing charge mobility and identify key factors that influence the material's dielectric performance, thereby providing valuable insights for optimizing energy storage applications.

Supplementary Note 9

Simulation of electronic energy level distributions

Molecular-scale simulations were employed to probe the localized effects of high-polarity dopants, which predominantly perturb the electronic structure and induce conformational changes in the immediate vicinity of adjacent polymer chains. Given this spatially confined influence, our GAUSSIAN-based framework offers an effective approach for capturing molecular-level electronic modifications. Additionally, the impact of dopants on the broadening of electronic energy levels was examined using density functional theory (DFT) calculations with the CAM-B3LYP functional and aug-cc-pVTZ basis set, enabling precise treatment of long-range electronic correlations and providing critical insights into the electronic disorder introduced by dopant incorporation. To broaden the scope of our analysis and capture long-range effects, we employed the high-precision aug-cc-pVTZ basis set, renowned for its accuracy in electronic structure calculations. To address the rapid decay of exchange interactions characteristic of conventional functionals, we utilized the CAM-B3LYP hybrid functional with long-range corrections, which provides a more precise treatment of electron exchange over extended distances. Furthermore, recognizing the critical yet often underappreciated role of dispersion forces in non-covalent interactions, we

incorporated Grimme's D3 empirical dispersion correction within the GAUSSIAN framework. This enhancement improves the accuracy of long-range dispersion interactions, thereby augmenting the overall reliability of our predictions for non-covalent interaction energies.

While a full implementation of CPA/ATA methodologies lies beyond the immediate scope of this study, we have adopted a suite of complementary computational strategies to approximate disorder-induced phenomena within the polymer composite system. To probe the effects of energetic disorder and complex polymer–dopant interactions, we employed supercell-based modeling within the VASP framework, enabling simulation of the associated broadening of electronic states in three dimensions.

To facilitate a comprehensive comparison, electronic energy level distributions were calculated using the Vienna Ab Initio Simulation Package (VASP), which employs a periodic framework to model the polymer composite in three dimensions. This approach effectively captures long-range interactions, which are essential for accurately describing the bulk electronic properties of the material. To mitigate artificial interactions, a real-space separation of 20 Å was introduced between periodic images, ensuring that the resulting properties closely resemble those of an ideal, infinite system. Concurrently, GAUSSIAN simulations were conducted on isolated PEI polymer chains, randomly doped with high-polarity dopants, to probe site-specific electronic perturbations. GAUSSIAN utilizes molecular orbital basis sets, including the widely validated aug-cc-pVTZ, to represent electron density via atomic orbitals, enabling a detailed and localized characterization of the electronic structure. Although the methodologies of VASP's periodic plane-wave pseudopotential approach and GAUSSIAN's non-periodic molecular orbital framework differ fundamentally, the electronic energy level distributions obtained from both methods show only minor discrepancies in absolute values, while preserving consistent overall trends. This agreement reinforces the robustness and reliability of the computational results.

Supplementary Note 10

Simulation of molecular dynamics

Molecular dynamics (MD) simulations were conducted using Orca 5.0.3 to investigate the effects of high-polarity small molecule doping on energy fluctuations. The simulations employed a timestep of 10 ps over a total duration of 2000 ps, performed at the B3LYP/def2-SVP level of theory with an initial temperature of 350 K. The ORCA MD module was utilized to provide molecular-level insights into the local interactions between polymer chains and high-polarity dopant molecules. This approach facilitates a detailed investigation of localized phenomena, including molecular conformations, dipole-dipole interactions, and short-range structural fluctuations within the polymer matrix. However, it is important to note that the absence of periodic boundary conditions in ORCA-based MD simulations limits their ability to capture long-range structural features, such as chain cross-linking and spatial heterogeneity, which are known to significantly influence the dielectric and charge transport properties of polymer composites. Consequently, ORCA was selected for its strengths in probing localized molecular-scale interactions that are vital for understanding short-range behavior, while periodic DFT simulations (via VASP) were employed to capture the complementary long-range phenomena. This dual-method approach ensures a comprehensive understanding of both local and extended structural characteristics within the polymer composite system.

We have expanded our computational analysis by conducting additional molecular dynamics (MD) simulations using the Vienna Ab initio Simulation Package (VASP). The implementation of periodic boundary conditions and the supercell approach within VASP enables a more accurate and holistic representation of the polymer composite system, capturing long-range electronic interactions and structural dynamics. These simulations provide deeper insights into the effects of polymer chain cross-linking on charge distribution and structural integrity, offering a more realistic depiction of the amorphous polymer matrix, including density fluctuations and packing effects. We utilized the Vienna Ab Initio Simulation Package (VASP) to conduct molecular dynamics (MD) simulations on a polymer composite system. These simulations were performed under periodic boundary conditions, enabling the modeling of the system in

three dimensions and the capture of long-range interactions, such as chain cross-linking and the effects of polymer chain packing on electronic properties. The system was modeled within a simulation box of 100×100×100 Å, and a buffer distance of 20 Å between atomic configurations and the box boundaries was maintained to mitigate finite-size effects and ensure adequate real-space separation. Electronic exchange-correlation interactions were described using the Perdew-Burke-Ernzerhof (PBE) functional within the generalized gradient approximation (GGA). A projector augmented wave (PAW) pseudopotential plane-wave basis set was employed to accurately model long-range interactions and delocalized charge distributions. Dispersion forces were included via the DFT-D3 correction scheme, which accounts for van der Waals interactions. The plane-wave energy cutoff was set to 500 eV to ensure a robust representation of the wavefunction, with an energy convergence criterion of 10^{-6} eV, ensuring high numerical precision.

A comparative analysis of the simulation results obtained from ORCA and VASP has implemented to assess their respective contributions and ensure the robustness of the findings. This analysis focuses on key aspects, including structural stability, energetic disorder, and intermolecular interactions, emphasizing differences in polymer chain conformation, dopant dispersion, and charge transport behavior between finite molecular models and periodic boundary conditions. While ORCA-based simulations offer valuable insights into localized molecular interactions, VASP simulations provide a more comprehensive representation of bulk properties and spatial heterogeneity within the polymer matrix. To complement these simulations, we compared the results with those from previous ORCA-based molecular modeling studies. ORCA, employing a quantum mechanics/molecular mechanics (QM/MM) hybrid approach, facilitated a more localized examination of molecular interactions, providing insights into short-range effects. The consistent trends observed between the results from both VASP and ORCA simulations serve to reinforce the reliability and robustness of the computational methodologies employed, thereby further validating the conclusions derived from the simulations. Additionally, MD simulations were utilized to examine the influence of doping molecules with large dipole moments on the conformational energy distribution

and free volume of polymers. Detailed models of the polymer and dopant molecules were constructed to assess the effects of varying doping concentrations. Analyses focused on the conformational energy distributions, elucidating the impact of dipole-dipole interactions on the polymer's structural landscape. The free volume of the doped polymer was also quantified using techniques such as Voronoi tessellation, illustrating how doping modifies the unoccupied spaces within the polymer matrix.

Supplementary Note 11

Phase-field model for electrical-thermal breakdown

The bipolar charge injection and transportation model are applied to simulate the complex process of charge injection and transportation within the polymer nanocomposites. The Schottky emission mechanism is utilized to describe the charge injection at the interface between the electrode and dielectric. The resulting current density at both the cathode and anode, denoted as $j_{c,a}$, can be determined as follows:

$$j_{c,a} = AT^2 \exp\left(-\frac{\varphi_{c,a}}{KT}\right)\exp\left(\frac{\sqrt{e^3 E_{c,a}/4\pi\varepsilon}}{KT}\right) \tag{S4}$$

Here, A is the Richardson coefficient, T stands for the temperature, $\varphi_{c,a}$ represents the charge injection barrier at the cathode and anode, k is the Boltzmann constant, e is the element charge, $E_{c,a}$ is the electric field at the cathode and anode, ε is the dielectric constant.

The dynamics of charge transport within the polymer nanocomposite are governed by the following set of equations.

$$\begin{cases} \dfrac{dn_e}{dt} + \nabla(-n_e\mu_e E - D_e\nabla n_e) = -R_{eh}n_e n_h - R_{eht}n_e n_{ht} - T_e n_e\left(1-\dfrac{n_{et}}{n_{0et}}\right) + v_e \exp\left(\dfrac{\phi_e}{KT}\right) n_{et}\dfrac{n_{et}}{n_{0et}} \\ \dfrac{dn_h}{dt} + \nabla(-n_h\mu_h E - D_h\nabla n_h) = -R_{eh}n_e n_h - R_{eth}n_{et} n_h - T_h n_h\left(1-\dfrac{n_{ht}}{n_{0ht}}\right) + v_h \exp\left(\dfrac{\phi_h}{KT}\right) n_{ht}\dfrac{n_{ht}}{n_{0ht}} \\ \dfrac{dn_{et}}{dt} = -R_{eth}n_{et}n_h - R_{etht}n_{et}n_{ht} + T_e n_e\left(1-\dfrac{n_{et}}{n_{0et}}\right) - v_e \exp\left(\dfrac{\phi_e}{KT}\right) n_{et}\dfrac{n_{et}}{n_{0et}} \\ \dfrac{dn_{ht}}{dt} = -R_{eht}n_e n_{ht} - R_{etht}n_{et}n_{ht} + T_h n_h\left(1-\dfrac{n_{ht}}{n_{0ht}}\right) - v_h \exp\left(\dfrac{\phi_h}{KT}\right) n_{ht}\dfrac{n_{ht}}{n_{0ht}} \end{cases} \tag{S5}$$

where E is the electric field, n_e is the density of free electrons, n_h is the density of free holes, n_{et} is the density of trapped electrons, n_{ht} is the density of trapped holes, n_{0et} is the maximum trap density of electrons, n_{0ht} is the maximum trap density of holes. μ_e is the mobility of free electrons, μ_h is the mobility of free holes, D_e is the diffusion factor of electrons, D_h is the diffusion factor of holes. R_{eh} is the recombination coefficient of

free electrons and free holes. R_{eht} is the recombination coefficient of trapped electrons and free holes, R_{etht} is the recombination coefficient of trapped electrons and free holes. T_e is the coefficient of electron trapping, T_h is the coefficient of hole trapping, v_e is the coefficient of electron trapping, v_h is the coefficient of electron trapping, ϕ_e and ϕ_h are the electron trap depth and hole trap depth determined by the DFT calculation, respectively.

The charge diffusion factor D and charge mobility μ can be written as,

$$D = \mu \frac{kT}{e}, \mu = \mu_0 \exp\left(\sqrt{\frac{e^3 E}{4\pi\varepsilon}} \Big/ kT \right) \quad \text{(S6)}$$

The electric potential, denoted as Φ, can be computed based on the distribution of free and trapped charge carrier densities through the following calculation.

$$\nabla^2 \Phi = -\frac{e\left(n_h + n_{ht} - n_e - n_{et}\right)}{\varepsilon} \quad \text{(S7)}$$

Upon solving the bipolar charge injection and transportation model, the derived electric field distribution is integrated into the phase field model to simulate the propagation of the breakdown phase. In this phase field model, a continuous phase-field variable denoted as $\eta(\mathbf{r}, t)$ is introduced to characterize the spatial and temporal evolution of the breakdown phase: $\eta(\mathbf{r}, t)$=1 signifies the breakdown phase, while $\eta(\mathbf{r}, t)$=0 signifies the non-breakdown phase. The free energy, denoted as F, is formulated by considering the collaborative influences from phase separation, interface effects, temperature, and electric field within an inhomogeneous dielectric, expressed as follows:

$$F = \int \left[f_{sep}(\eta(r)) + f_{grad}(\eta(r)) + f_{elec}(\eta(r)) + f_{Joule}(\eta(r)) + f_{strain}(\eta(r)) \right] dV \quad \text{(S9)}$$

where f_{sep} is the free energy density of mixing the drives the phase separation, f_{grad} is the gradient energy density, f_{elec} is the electrostatic energy density, f_{Joule} is the thermal energy density, and f_{strain} is the strain energy density. The evolution of the breakdown phase is delineated using a modified Allen-Cahn equation.

$$\frac{\partial \eta(r,t)}{\partial t} = -L_0 H(f_{elec} + f_{Joule} + f_{strain}) \left[\frac{\partial f_{sep}(\eta)}{\partial \eta(r,t)} + \frac{\partial f_{grad}(\eta)}{\partial \eta(r,t)} + \frac{\partial f_{elec}(\eta)}{\partial \eta(r,t)} + \frac{\partial f_{Joule}(\eta)}{\partial \eta(r,t)} + \frac{\partial f_{strain}(\eta)}{\partial \eta(r,t)} \right] \quad \text{(S10)}$$

where L_0 is the kinetic coefficient relating to the interface mobility with a value of 1 m^2 s^{-1} N^{-1}, and H is the Heaviside unit step function.

Supplementary Note 12

Fabrication of large-area films

The preparation of high-quality, large-area composite films via the solution-casting method involves several critical steps to ensure consistency and desirable properties across the films. The process begins with the synthesis of polyamic acid (PAA), the precursor to polyimide, through the reaction of dianhydride and diamine monomers in a solvent such as N,N-dimethylacetamide (DMAc). This PAA solution is meticulously mixed to achieve a homogeneous and viscous solution. The solution is then cast onto a moving belt, with the initial layer's thickness carefully controlled by adjusting factors such as solution viscosity, casting speed, and the gap of the doctor blade or other casting equipment. After casting, the substrate with the wet PAA layer passes through a series of drying chambers or ovens, where the solvent is gradually evaporated under controlled heating conditions. Concurrently, the polyamic acid undergoes thermal imidization, converting it into polyimide. Once imidization is complete, the resulting polyimide film is gently peeled from the substrate. In certain cases, additional post-treatment steps, such as surface modification, stretching, or annealing, may be applied to further enhance specific film properties.

Supplementary Note 13

Conduction mechanism

Hopping conduction describes the mechanism by which trapped electrons move between various trap sites within dielectric films, a process facilitated by tunneling effects. The resultant current density associated with hopping conduction can be mathematically expressed as follows:

$$J = 2nq\lambda v \exp\left(-\frac{E_a}{k_B T}\right)\sinh\left(\frac{\lambda q E}{2k_B T}\right) \tag{S11}$$

Here, n signifies the carrier density, n represents the electric charge of the carriers, λ denotes the average hopping distance, v stands for the attempt-to-escape frequency, E_a characterizes the activation energy associated with hopping conductance, k_B symbolizes the Boltzmann constant, T signifies the absolute temperature, and E represents the electric field strength. The above equation can be simplified as

$$J = J_0 \times \sinh(\alpha E) \tag{S12}$$

Where J_0 and α represent two lumped parameters. Consequently, we can model the ln(J) versus E curves using hopping conduction and determine the hopping distance, which is included in parameter α.

Supplementary Note 14

Dielectric breakdown behavior

The breakdown strength (E_b) of PEI composites was assessed using a two-parameter Weibull statistical analysis, described as follows:

$$P(E) = 1 - \exp\left(-\left(\frac{E}{E_b}\right)^{\beta}\right) \tag{S13}$$

Where P is the probability of breakdown failure, E is the experimentally measured electric breakdown field, E_b is the Weibull breakdown strength at a 63.2% probability of breakdown, and β is a shape parameter that evaluates the data scatter.

Supplementary Note 15

Storage energy density

The energy storage density U_e of dielectric materials is governed by the relationship between electric displacement D and the applied electric field E, and is quantitatively described by the following relationship:

$$U_e = \int_0^D E dD \tag{S14}$$

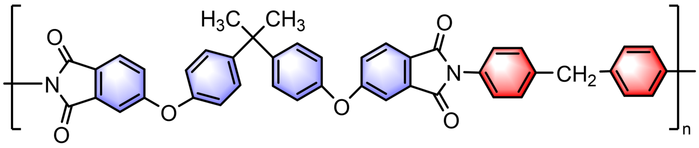

Supplementary Figure S1. Chemical structure of the PEI used in this work.

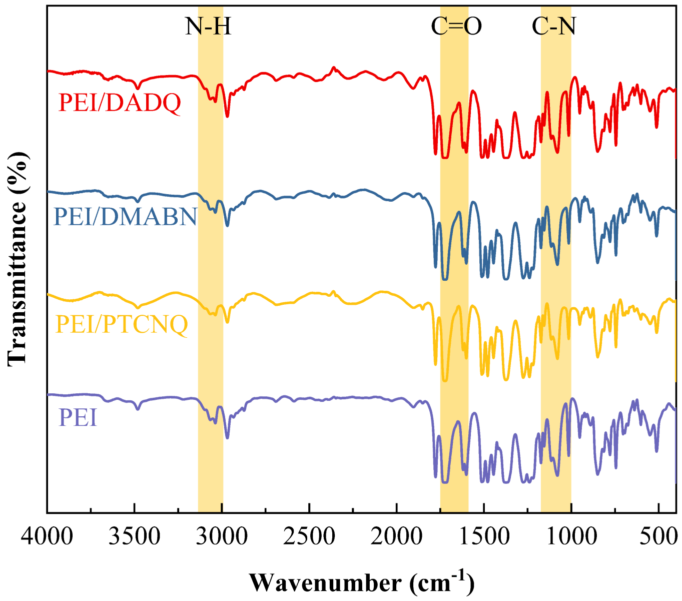

Supplementary Figure S2. FTIR spectra of pristine PEI, PEI/DADQ, PEI/DMABN and PEI/PTCNQ composite films.

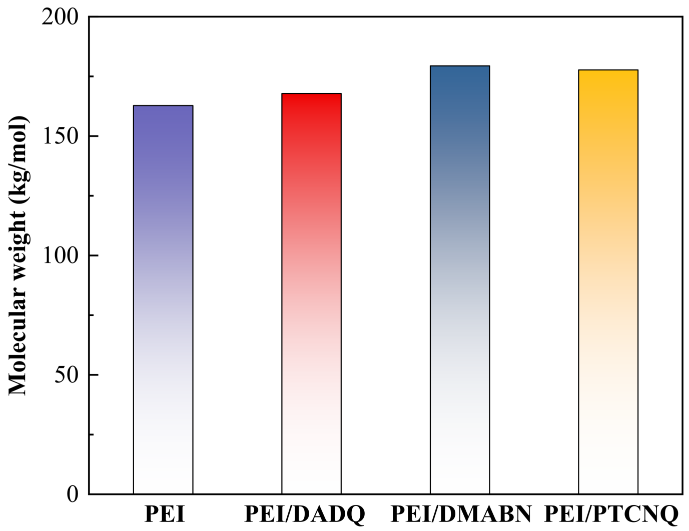

Supplementary Figure S3. Molecular weight from gel permeation chromatography (GPC) of pristine PEI, PEI/DADQ, PEI/DMABN and PEI/PTCNQ.

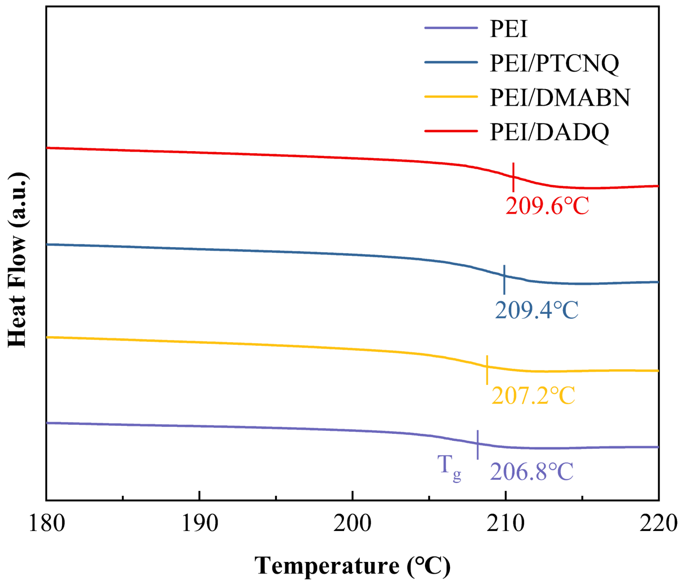

Supplementary Figure S4. Glass transition temperature (T_g) determined from differential scanning calorimetry (DSC) curves of pristine PEI, PEI/DADQ, PEI/DMABN and PEI/PTCNQ composite films.

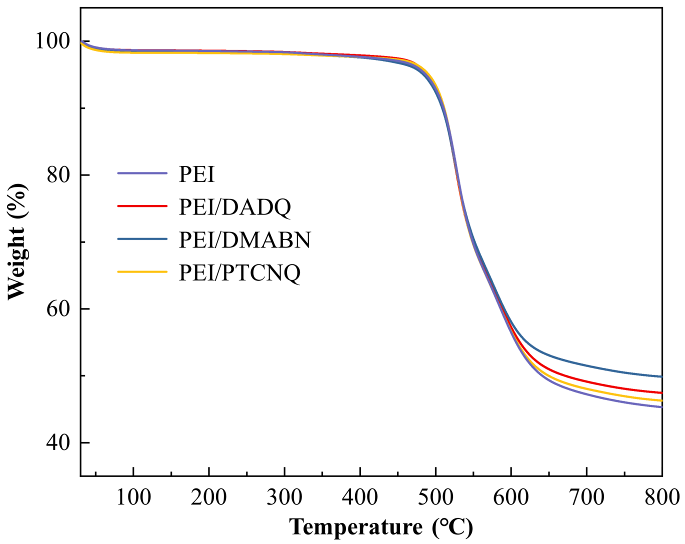

Supplementary Figure S5. Thermogravimetric analysis curves of pristine PEI, PEI/DADQ, PEI/DMABN and PEI/PTCNQ composite films.

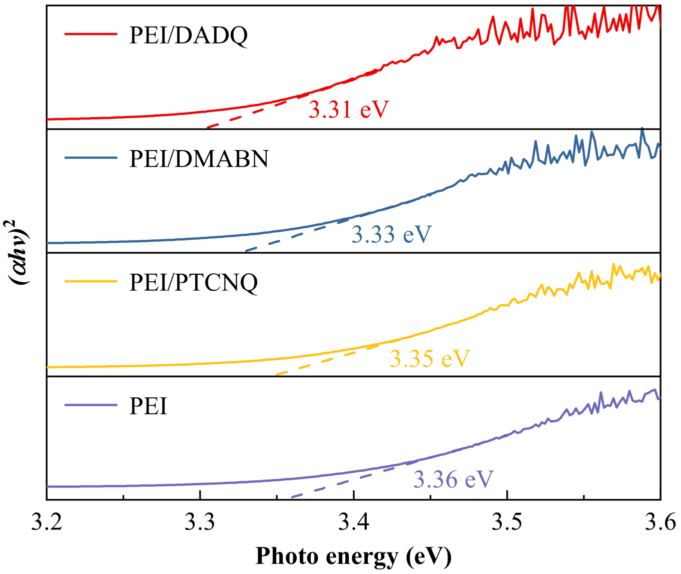

Supplementary Figure S6. UV-vis absorption spectra of pristine PEI, PEI/DADQ, PEI/DMABN and PEI/PTCNQ composite films.

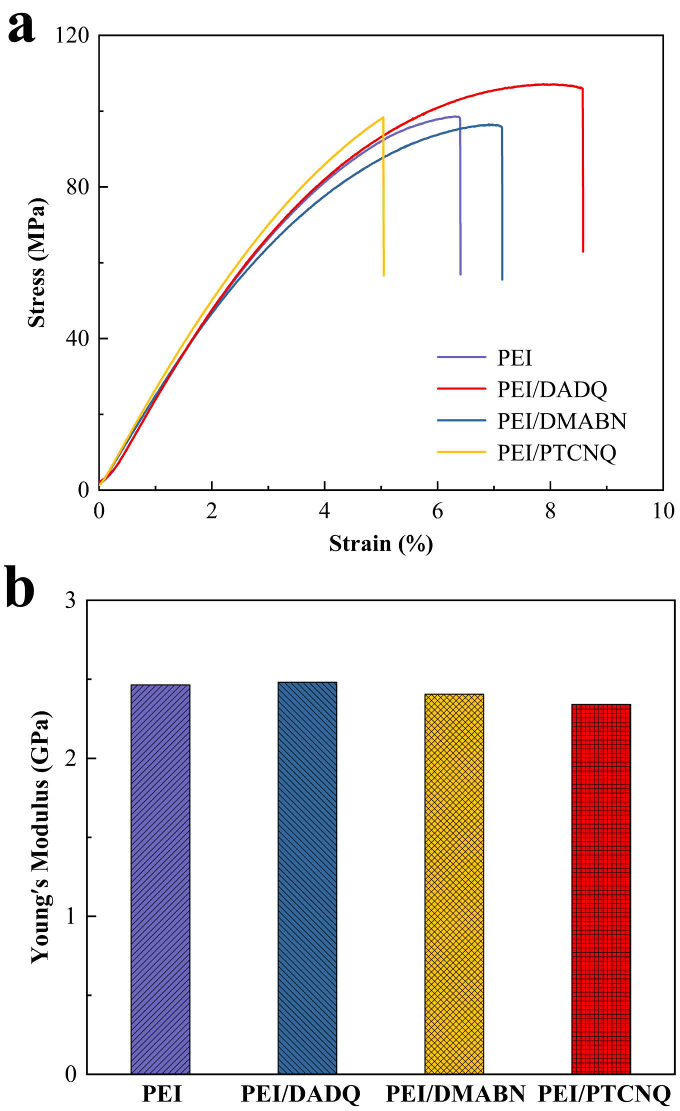

Supplementary Figure S7. (a) Stress strain curve and (b) Young's modulus of pristine PEI, PEI/DADQ, PEI/DMABN and PEI/PTCNQ composite films.

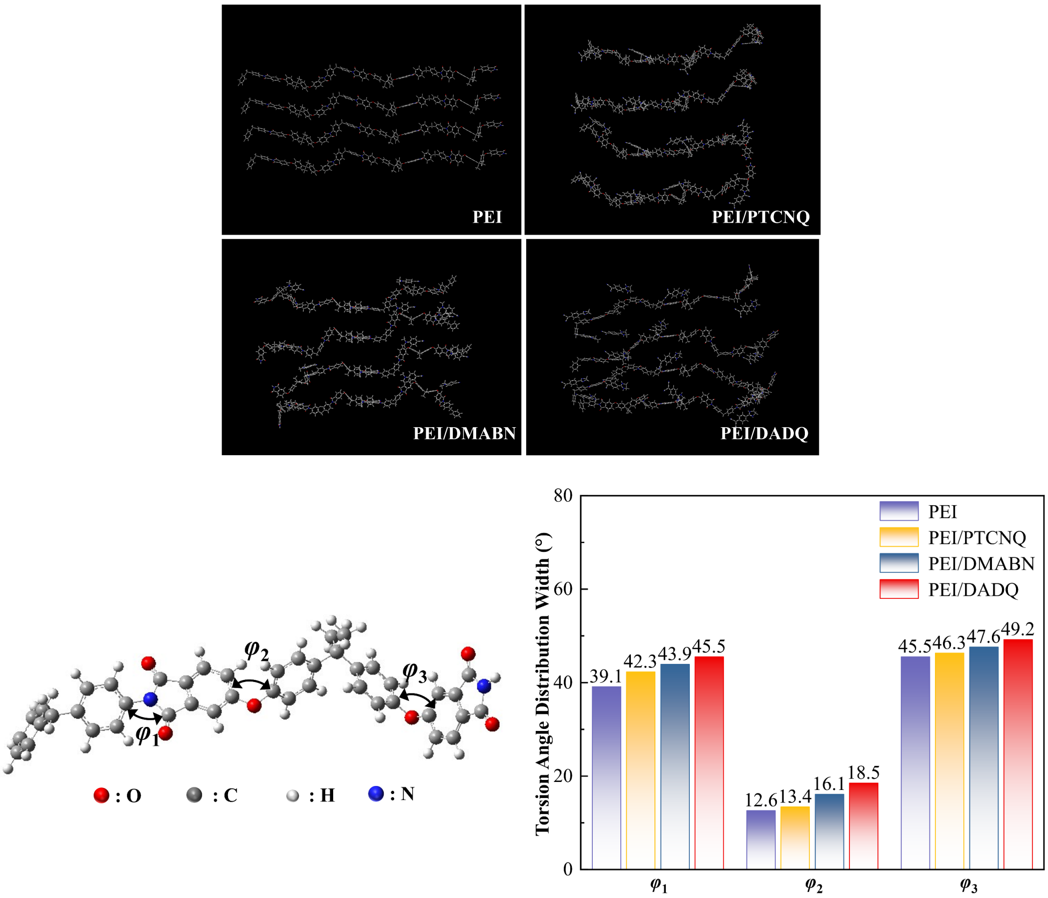

Supplementary Figure S8. Angular Distribution Width (W) of Polymer Backbone Torsion in Pristine PEI, PEI/DADQ, PEI/DMABN, and PEI/PTCNQ from Molecular Dynamics Simulations

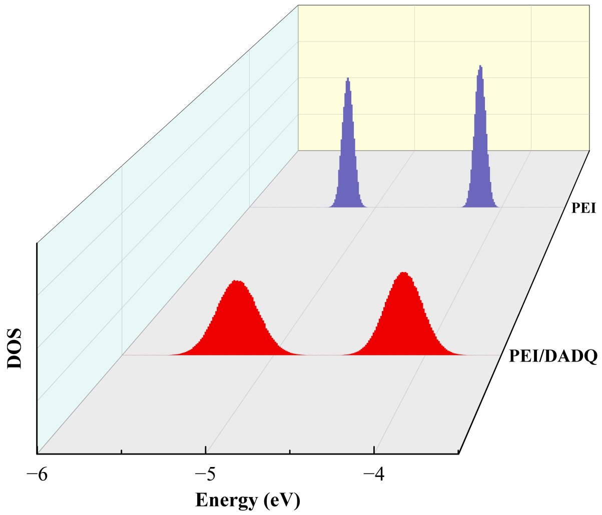

Supplementary Figure S9. Analysis of Electronic Energy Level Distributions in pristine PEI and PEI/DADQ composites using VASP

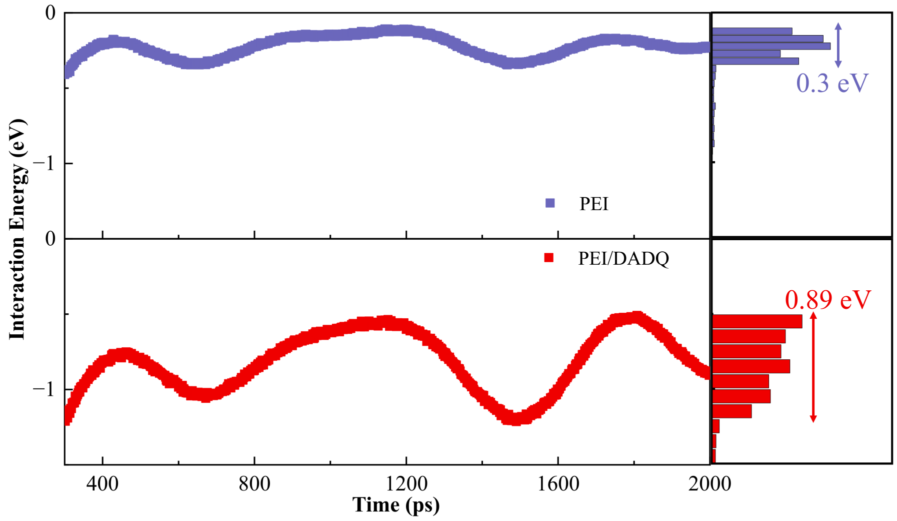

Supplementary Figure S10. Analysis of Interaction Energy Fluctuations in Pristine PEI and PEI/DADQ Composites via VASP.

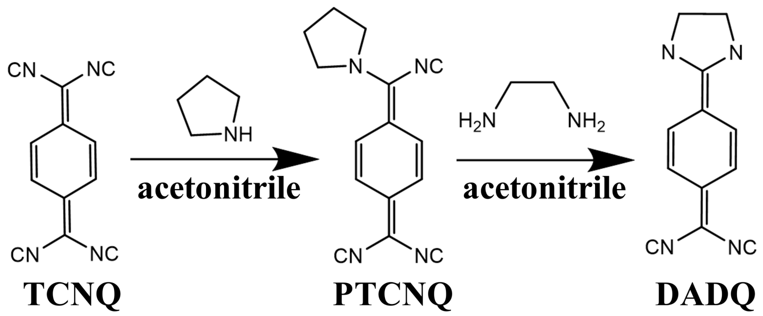

Supplementary Figure S11. Synthetic route detailing the activation of TCNQ via its reaction with pyrrolidine, leading to the formation of PTCNQ and DADQ.

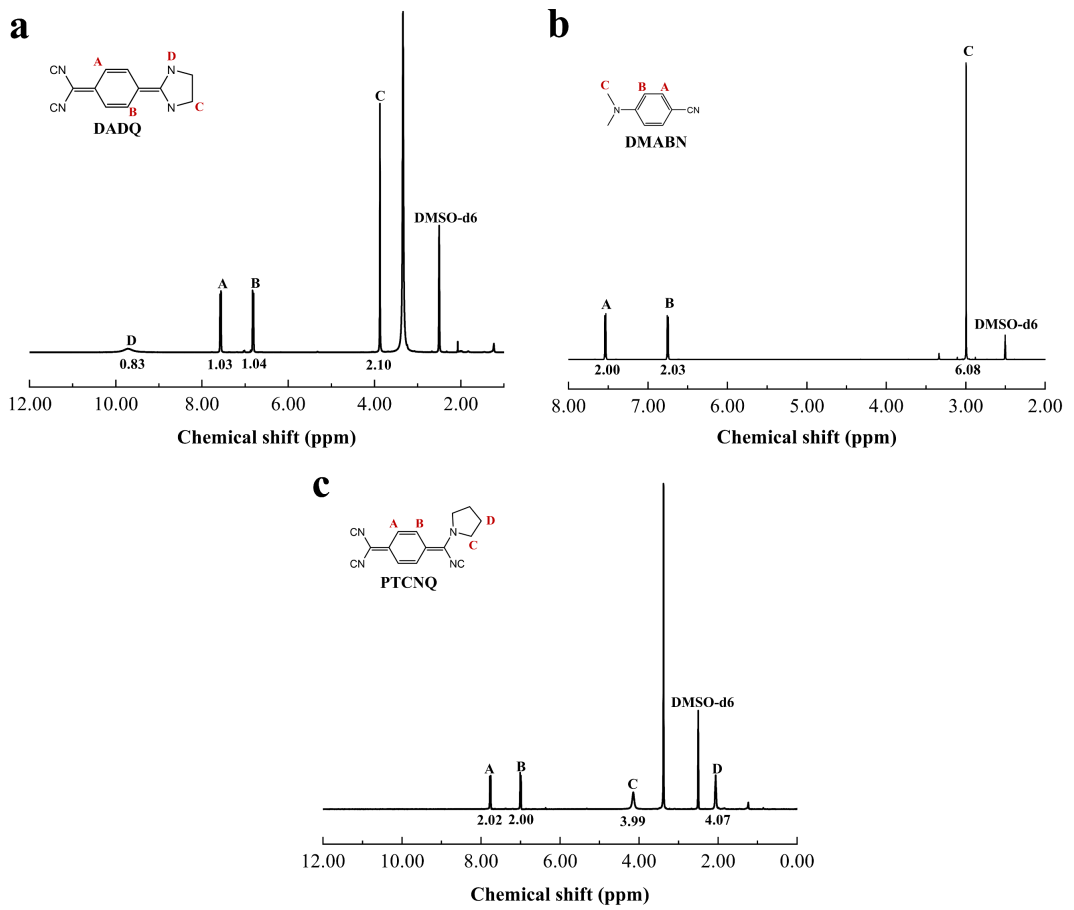

Supplementary Figure S12. ^{1}H NMR spectra of (a) DADQ, (b) DMABN and (c) PTCNQ.

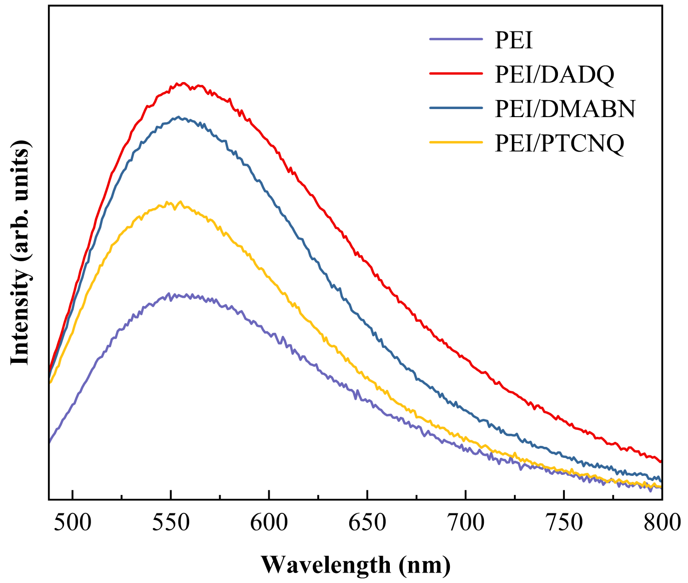

Supplementary Figure S13. Steady-state photoluminescence of pristine PEI, PEI/DADQ, PEI/DMABN and PEI/PTCNQ.

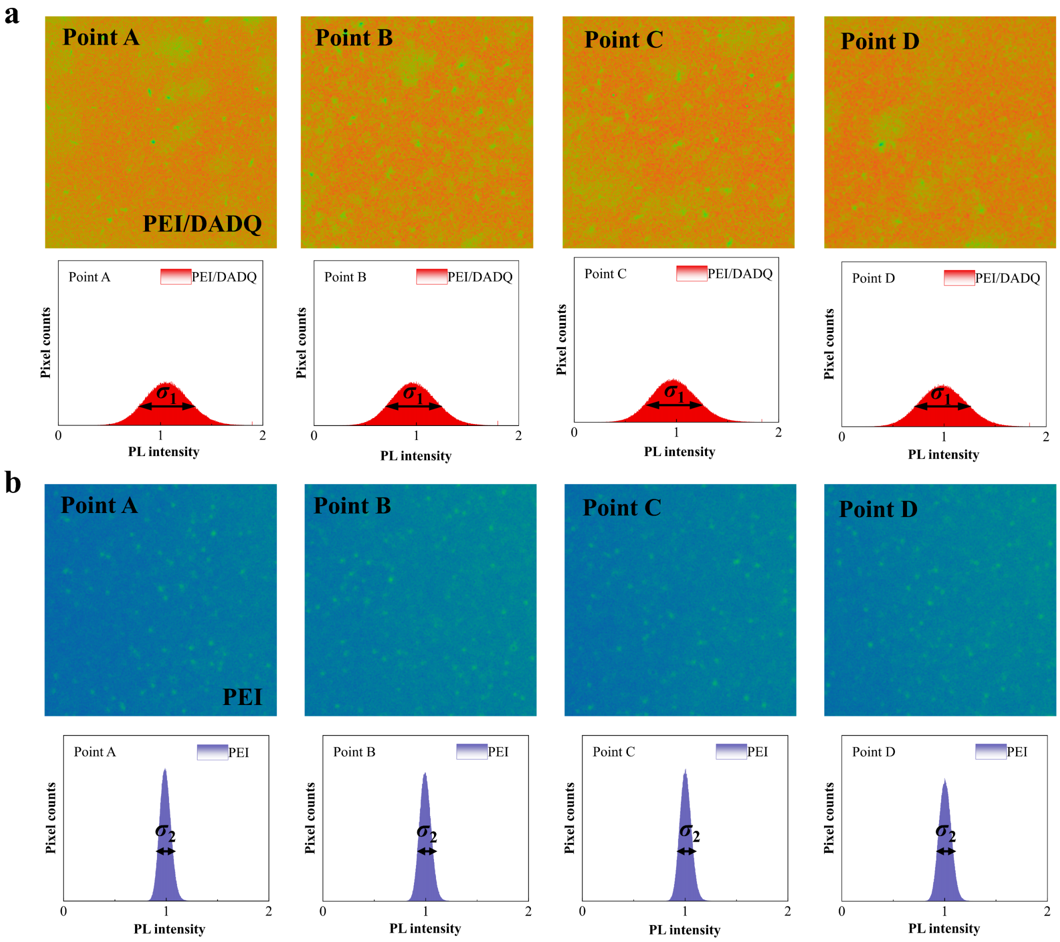

Supplementary Figure S14. Wide-field PL images of (a) PEI/DADQ and (b) pristine PEI at four distinct points under excitation at 405 nm.

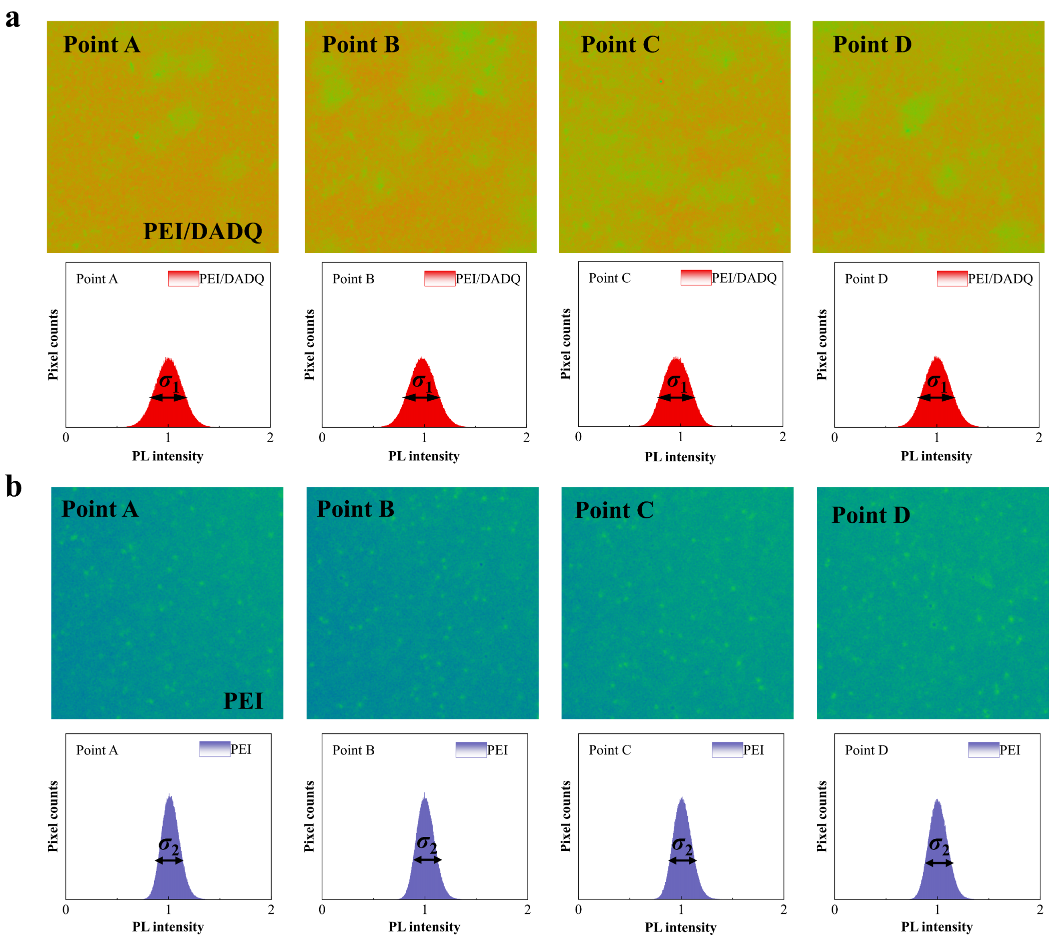

Supplementary Figure S15. Wide-field PL images of (a) PEI/DADQ and (b) pristine PEI at four distinct points under excitation at 488 nm.

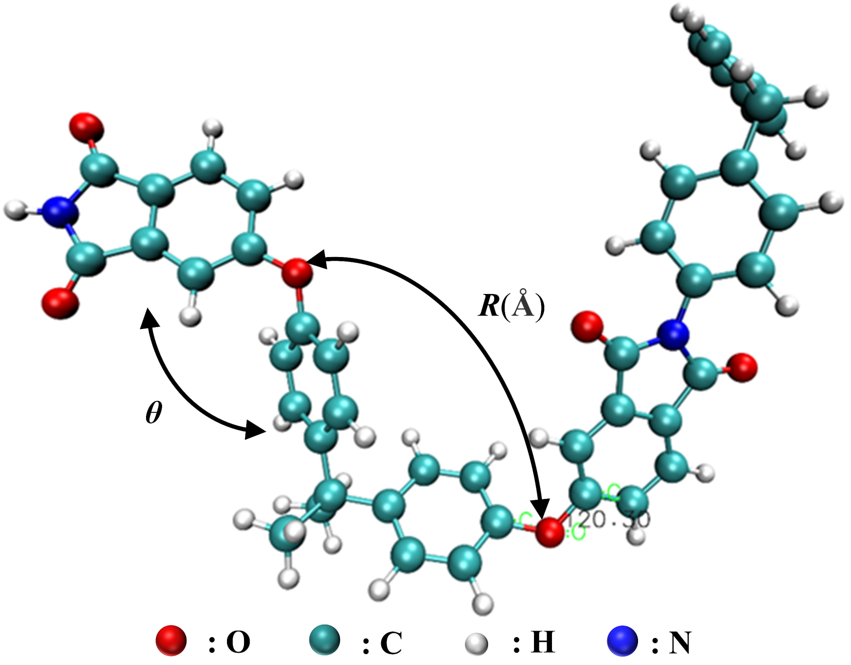

Supplementary Figure S16. Conformational structure of PEI chain

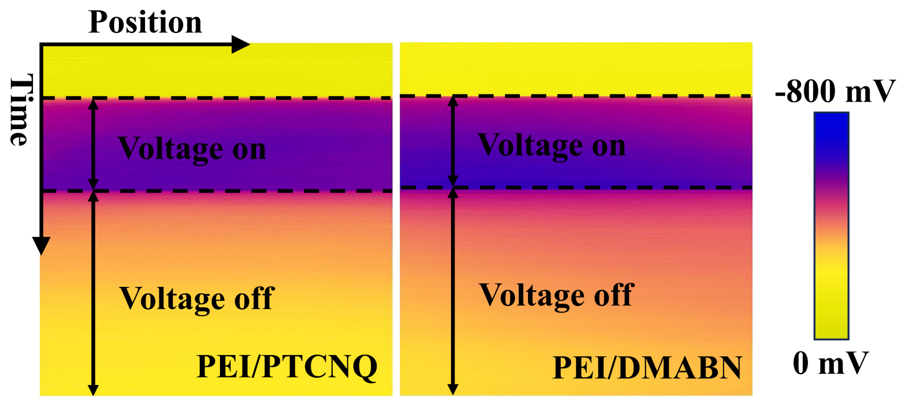

Supplementary Figure S17. Mapped time-dependent surface potential distribution on the scanned line in PEI/PTCNQ and PEI/DMABN.

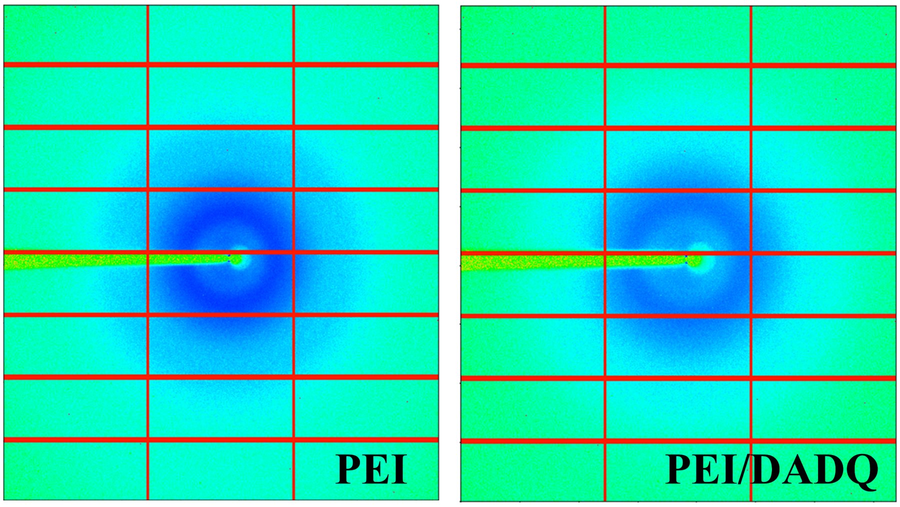

Supplementary Figure S18. 2D-WAXD patterns of pristine PEI and PEI/DADQ.

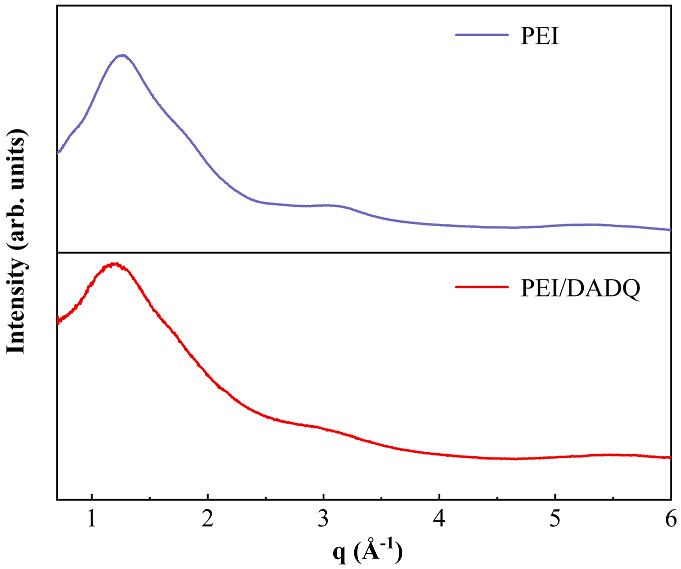

Supplementary Figure S19. 1D-WAXD profiles of pristine PEI and PEI/DADQ.

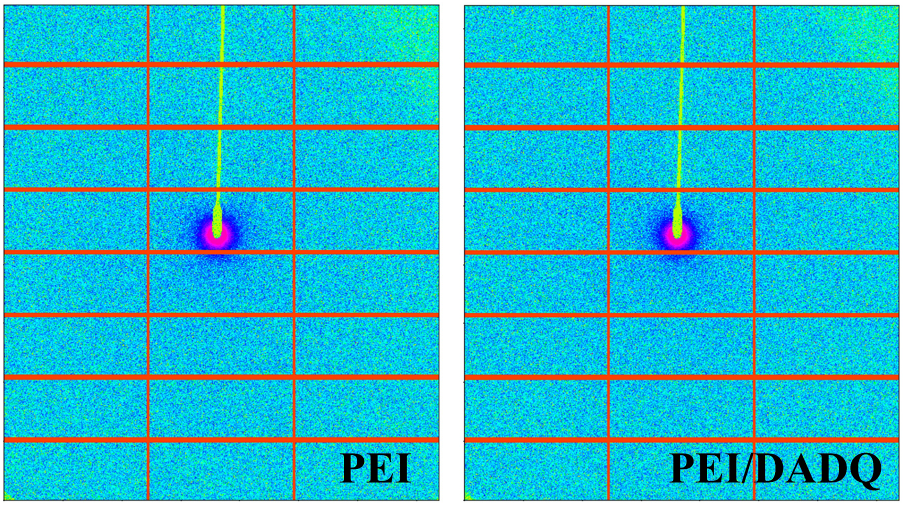

Supplementary Figure S20. 2D-SAXS patterns of pristine PEI and PEI/DADQ.

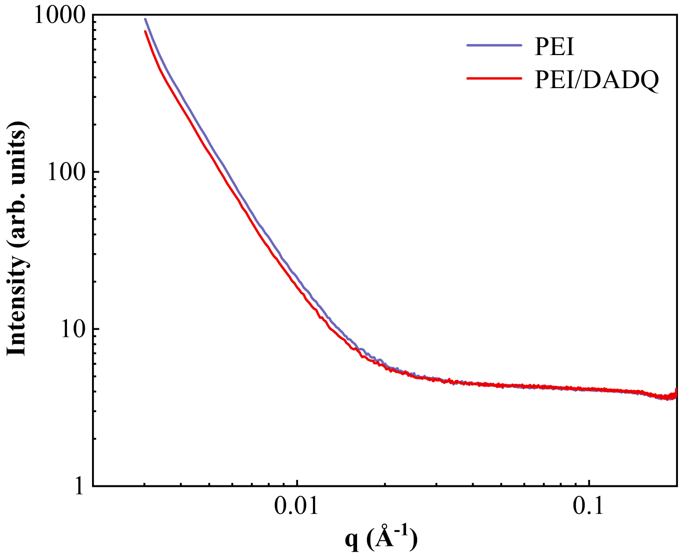

Supplementary Figure S21. 1D-SAXS profiles of pristine PEI and PEI/DADQ.

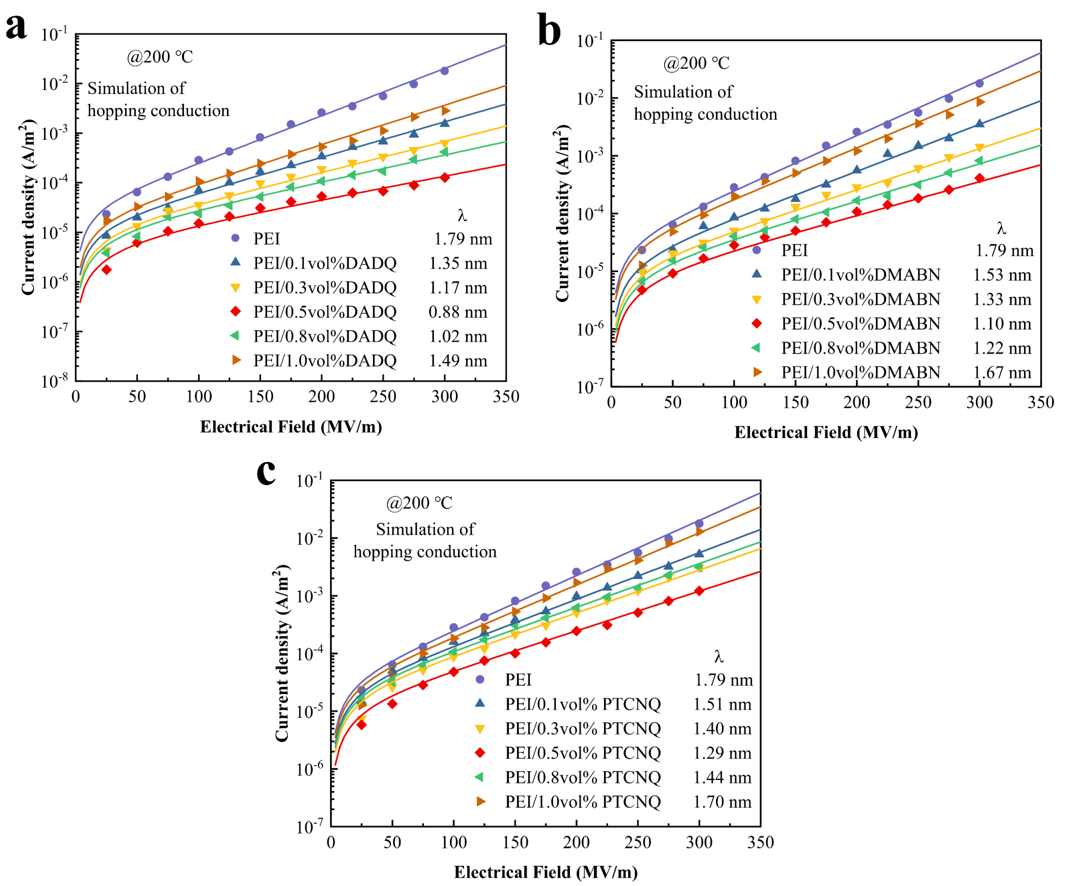

Supplementary Figure S22. Electric field-dependent conduction current density and charge carrier hopping distance of (a) PEI/DADQ, (b) PEI/DMABN and (c) PEI/PTCNQ at 200 °C.

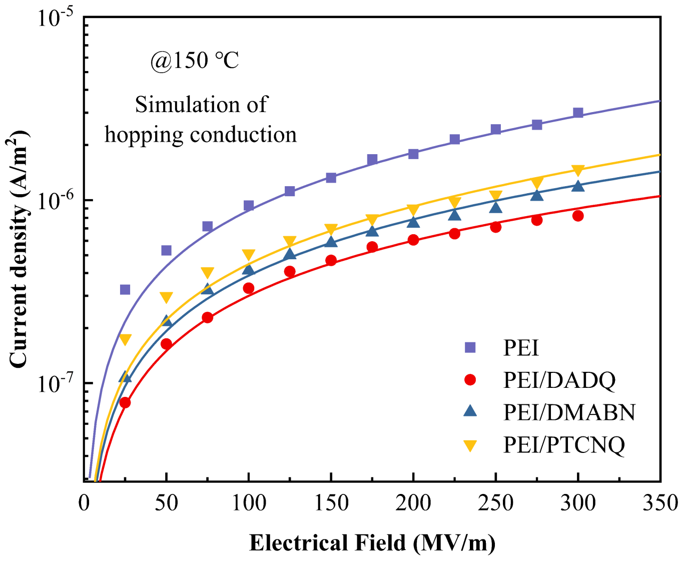

Supplementary Figure S23. Electric field-dependent conduction current density and charge carrier hopping distance of pristine PEI, PEI/DADQ, PEI/DMABN and PEI/PTCNQ at 150 °C.

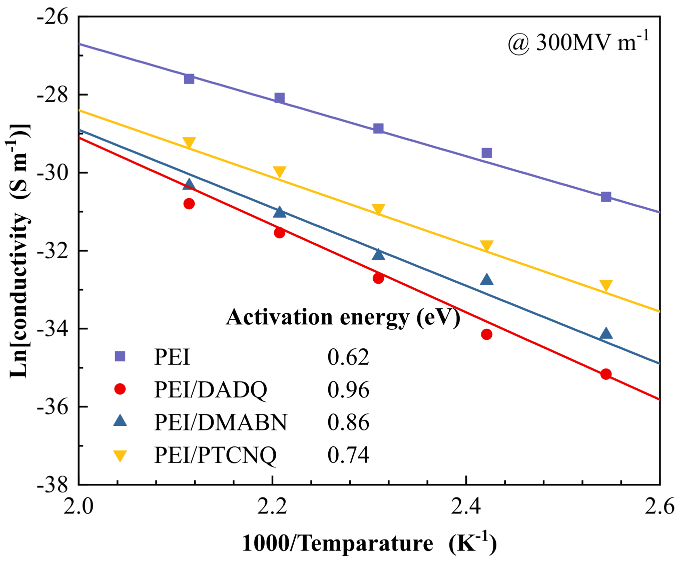

Supplementary Figure S24. Temperature-dependent electrical conductivity for pristine PEI, PEI/DADQ, PEI/DMABN and PEI/PTCNQ at 300 MV m^{-1}.

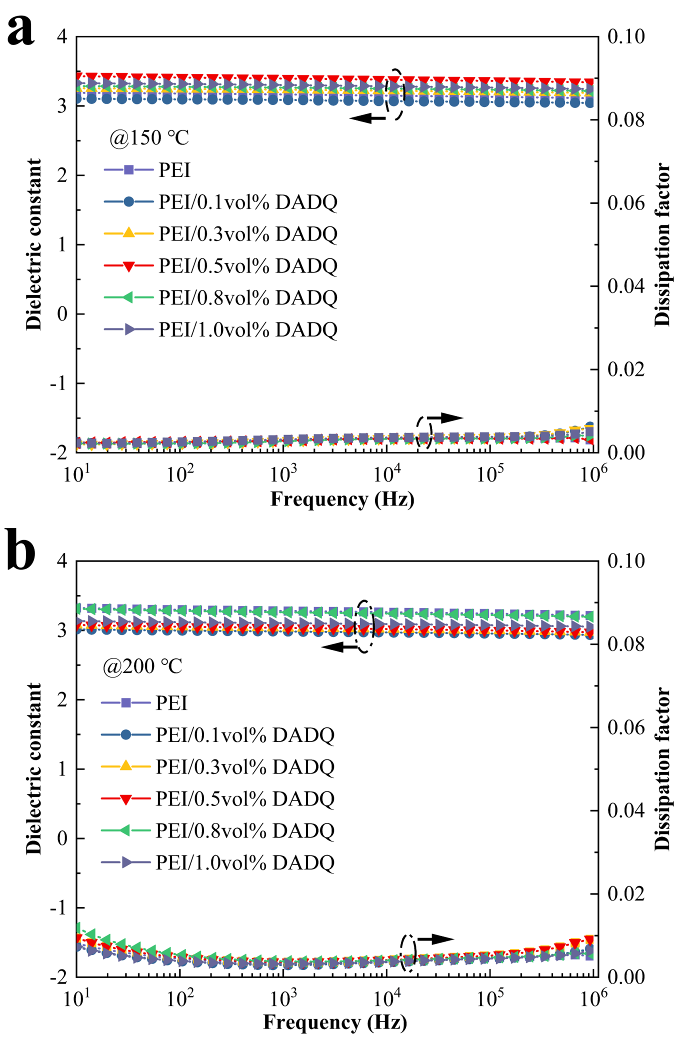

Supplementary Figure S25. Frequency-dependent dielectric constant and dissipation factor of PEI/DADQ composite with various DADQ contents at (a) 150 °C and (b) 200 °C.

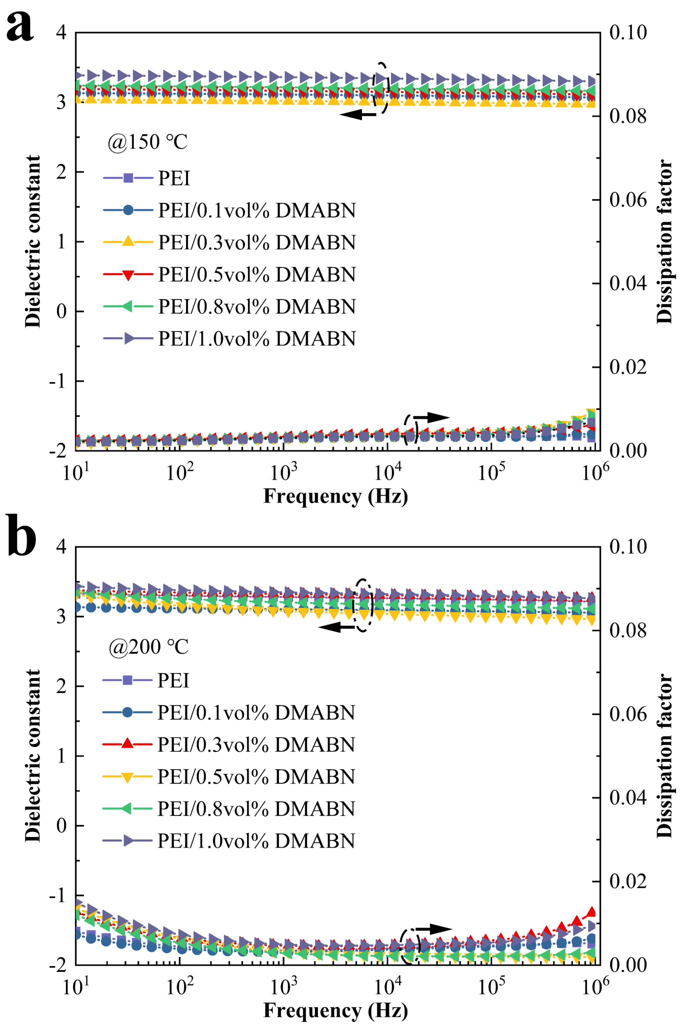

Supplementary Figure S26. Frequency-dependent dielectric constant and dissipation factor of PEI/DMABN composite with various DMABN contents at (a) 150 °C and (b) 200 °C.

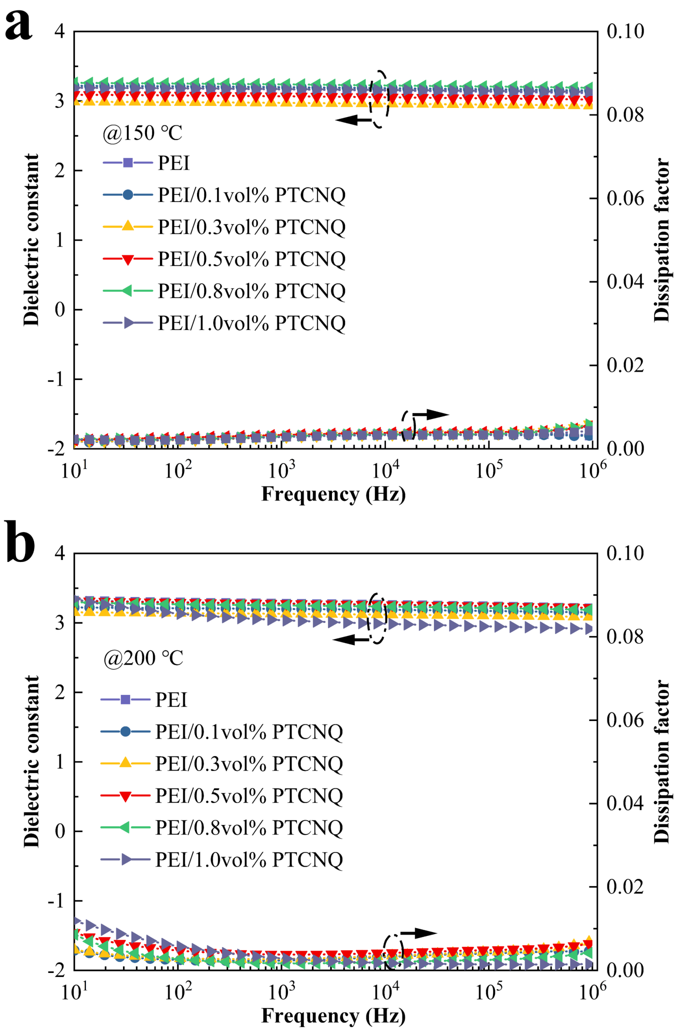

Supplementary Figure S27. Frequency-dependent dielectric constant and dissipation factor of PEI/PTCNQ composite with various PTCNQ contents at (a) 150 °C and (b) 200 °C.

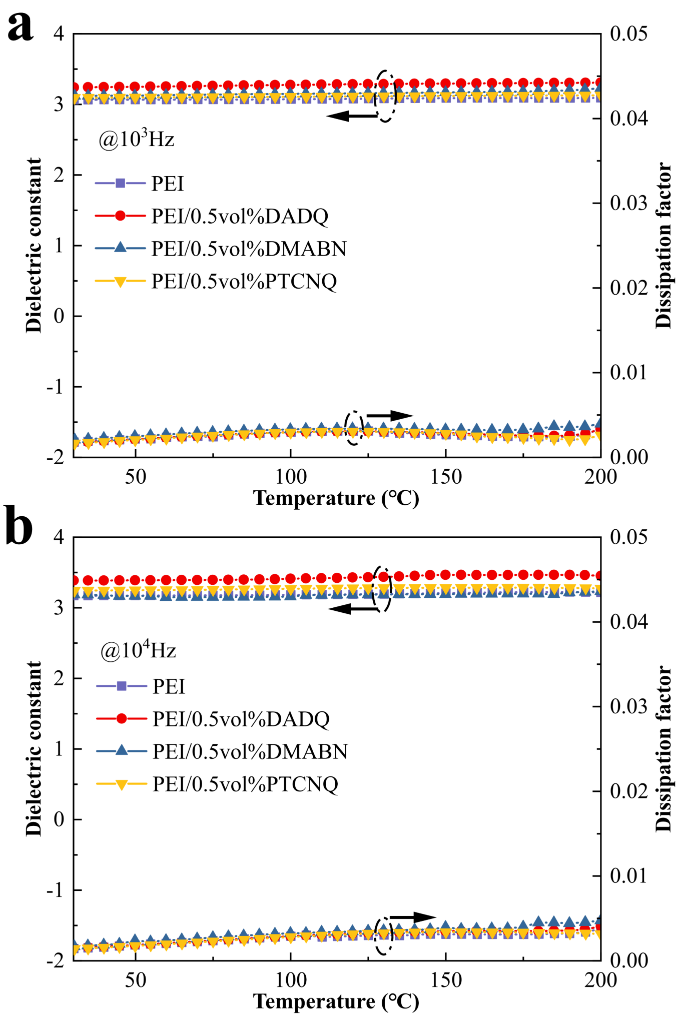

Supplementary Figure S28. Temperature-dependent dielectric constant and dissipation factor of pristine PEI, PEI/DADQ, PEI/DMABN and PEI/PTCNQ composite films at (a) 10^3 Hz and (b) 10^4 Hz.

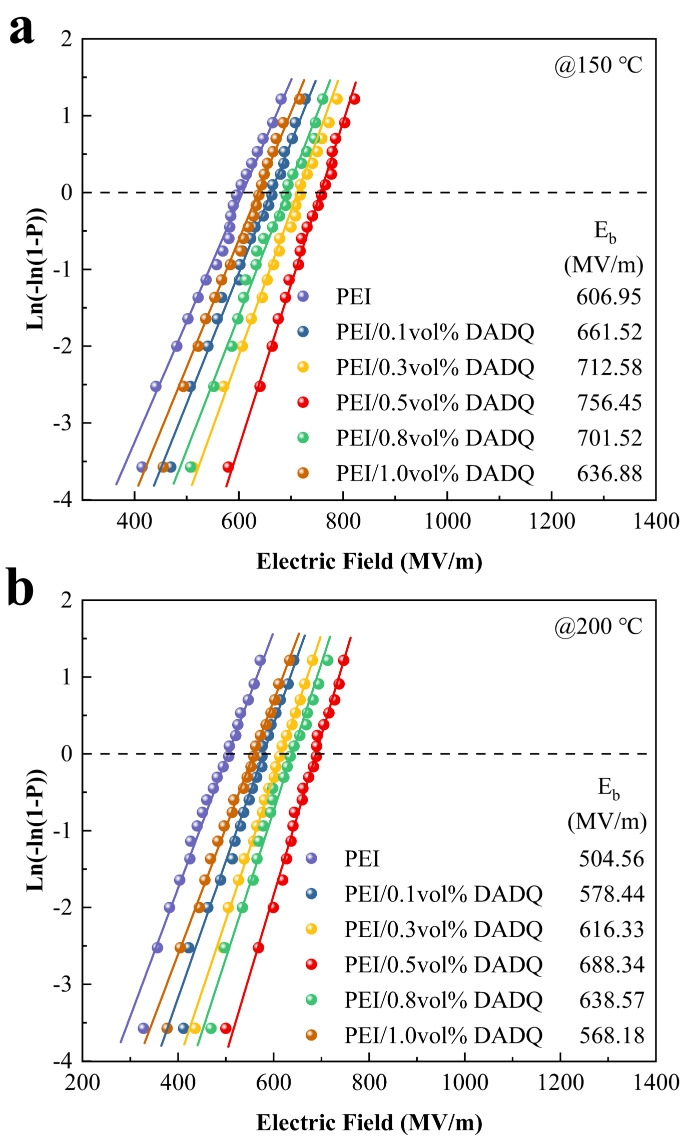

Supplementary Figure S29. Weibull plots of PEI/DADQ with various DADQ contents at (a) 150 °C and (b) 200 °C.

Supplementary Table S1. Weibull statistics of breakdown strength for the PEI/DADQ composite film as a function of volume of DADQ at 150 °C and 200 °C.

	**Volume Content**	0	0.1 %	0.3 %	0.5 %	0.8 %	1.0 %
**150 °C**	E_b MV/m	606.95	661.52	712.58	756.45	701.52	636.88
	β	9.16	10.3	11.22	14.23	11.24	10.36
**200 °C**	E_b MV/m	504.56	578.44	616.33	688.34	638.57	568.18
	β	7.95	9.76	10.78	12.56	10.94	8.63

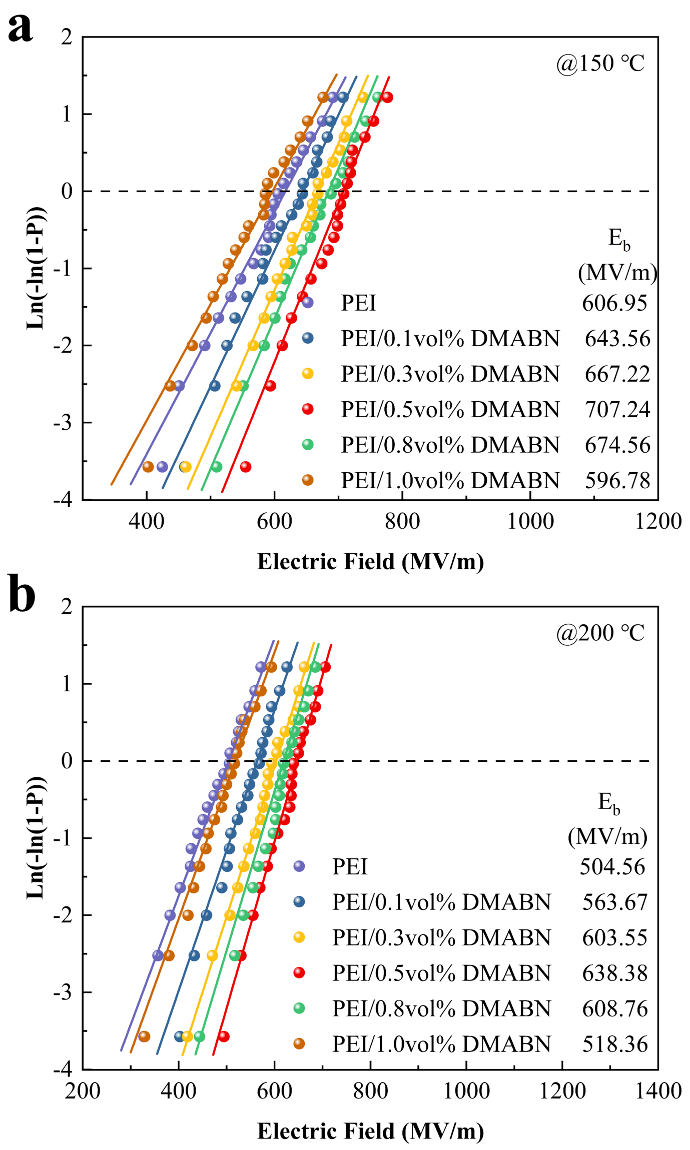

Supplementary Figure S30. Weibull plots of PEI/DMABN with various DMABN contents at (a) 150 °C and (b) 200 °C.

Supplementary Table S2. Weibull statistics of breakdown strength for the PEI/DMABN composite film as a function of volume of DMABN at 150 °C and 200 °C.

	**Volume Content**	0	0.1 %	0.3 %	0.5 %	0.8 %	1.0 %
**150 °C**	E_b MV/m	606.95	643.56	667.22	707.24	674.56	596.78
	β	9.16	10.64	10.81	13.63	11.65	8.96
**200 °C**	E_b MV/m	504.56	563.67	603.55	638.38	588.76	528.36
	β	7.95	10.47	10.52	11.45	10.77	8.48

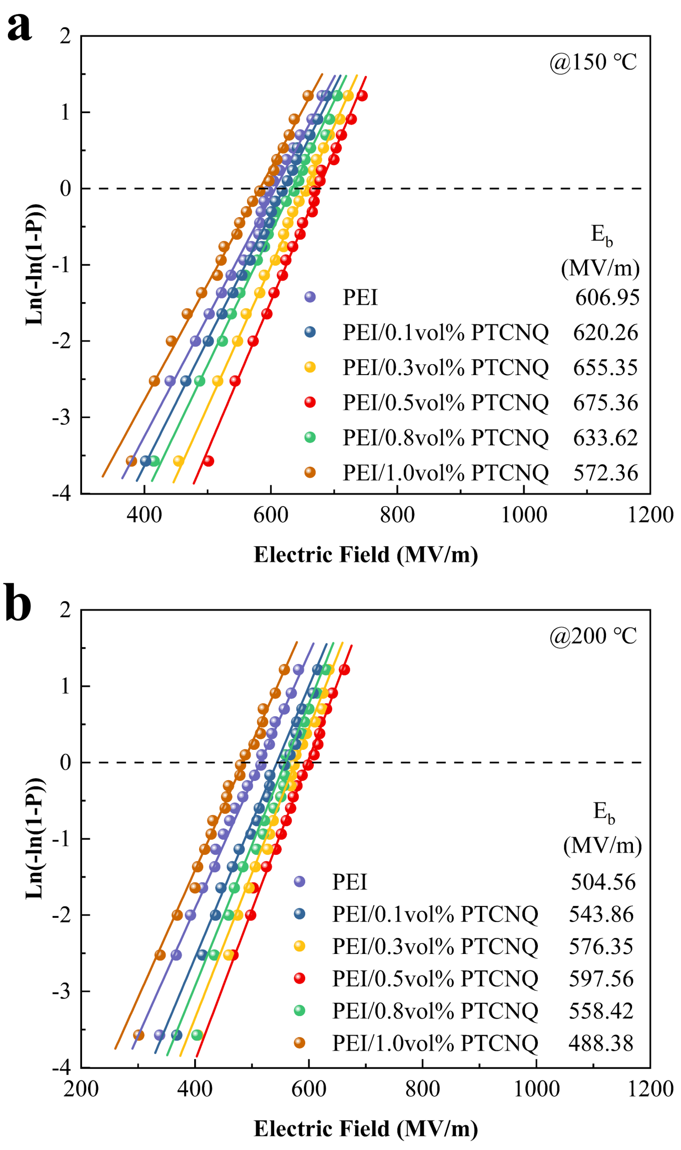

Supplementary Figure S31. Weibull plots of PEI/PTCNQ with various PTCNQ contents at (a) 150 °C and (b) 200 °C.

Supplementary Table S3. Weibull statistics of breakdown strength for the PEI/PTCNQ composite film as a function of volume of PTCNQ at 150 °C and 200 °C.

	**Volume Content**	0	0.1 %	0.3 %	0.5 %	0.8 %	1.0 %
**150 °C**	E_b MV/m	606.95	620.26	655.35	675.36	633.62	572.36
	β	9.16	9.07	10.56	12.14	9.28	8.09
**200 °C**	E_b MV/m	504.56	543.86	576.35	597.56	558.42	488.38
	β	7.95	8.75	10.53	10.2	10.14	7.58

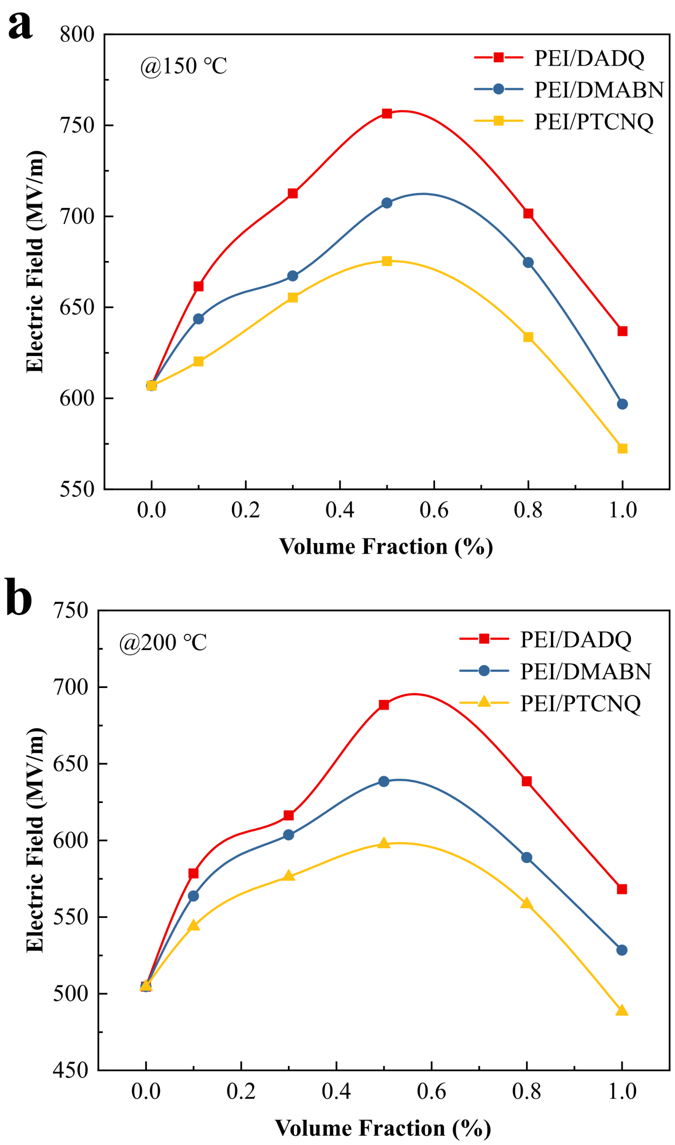

Supplementary Figure S32. Weibull breakdown strength versus volume fraction of PEI/DADQ, PEI/DMABN and PEI/PTCNQ composite films at (a) 150 ℃ and (b) 200 ℃.

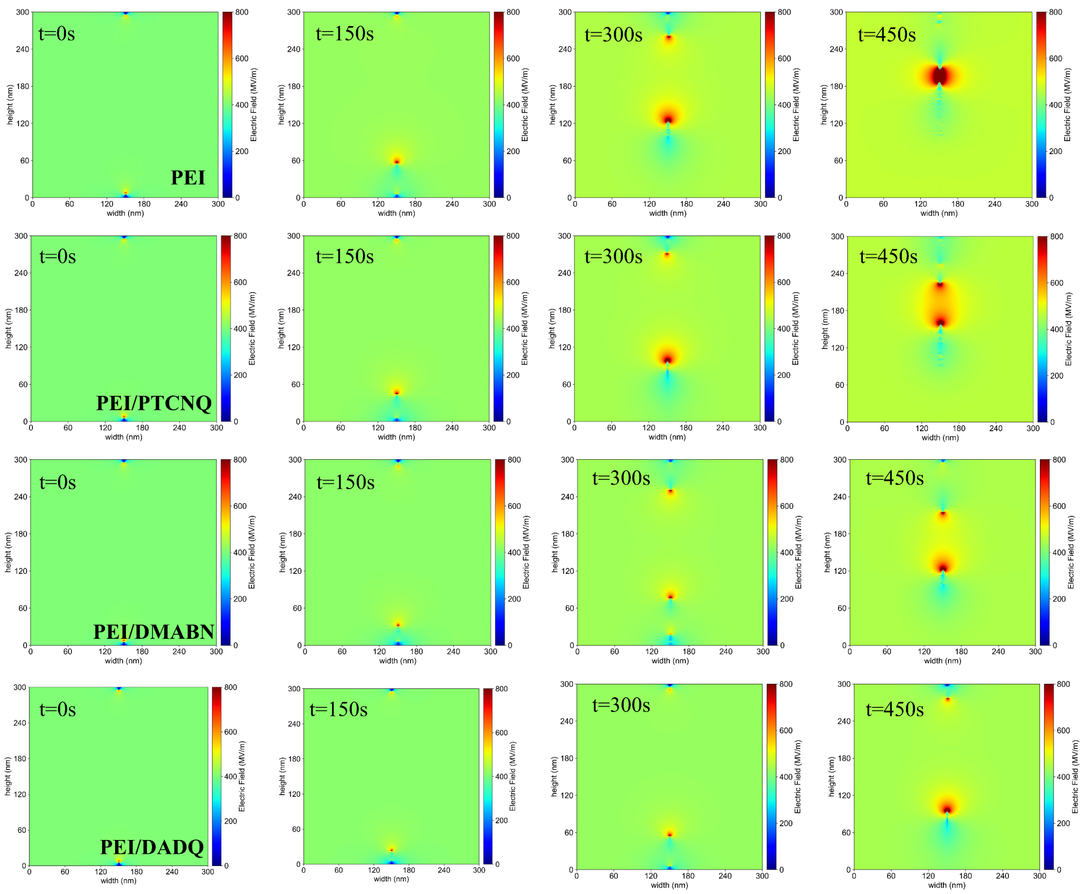

Supplementary Figure S33. Electric field distribution and breakdown phase propagation of pristine PEI, PEI/DADQ, PEI/DMABN and PEI/PTCNQ composite

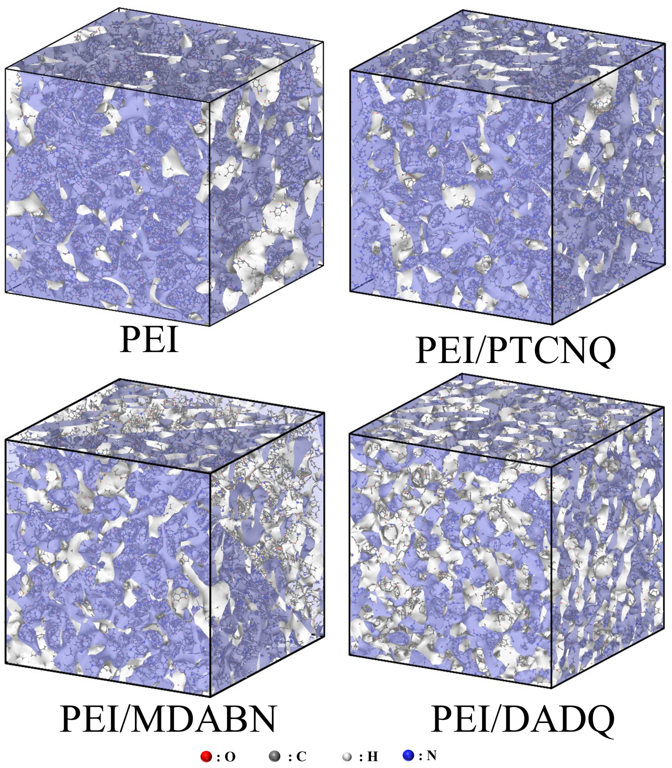

Supplementary Figure S34. Polymer configurations of pristine PEI, PEI/DADQ, PEI/DMABN and PEI/PTCNQ composite films obtained in molecular dynamics simulation.

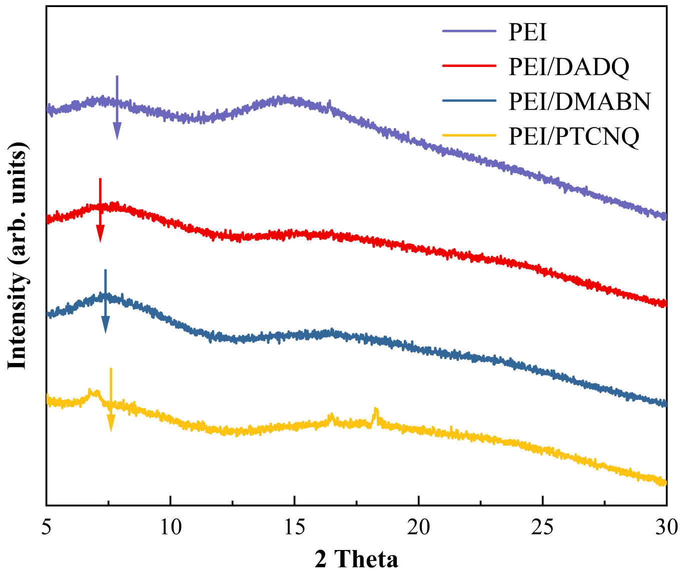

Supplementary Figure S35. of pristine PEI, PEI/DADQ, PEI/DMABN and PEI/PTCNQ composite films.

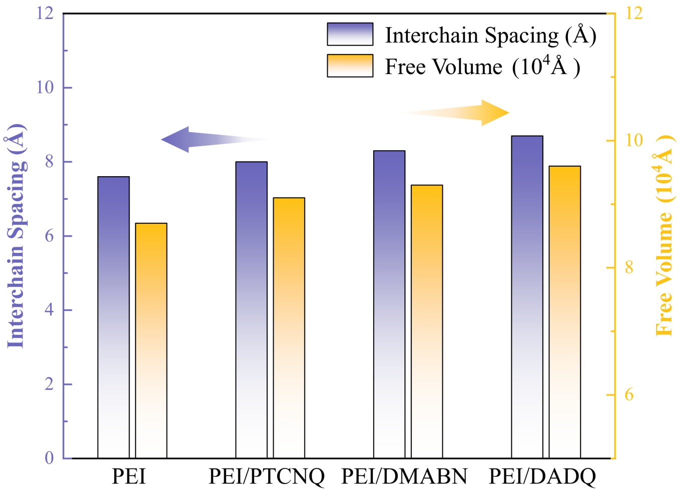

Supplementary Figure S36. Free volume and interchain spacing of pristine PEI, PEI/DADQ, PEI/DMABN and PEI/PTCNQ composite

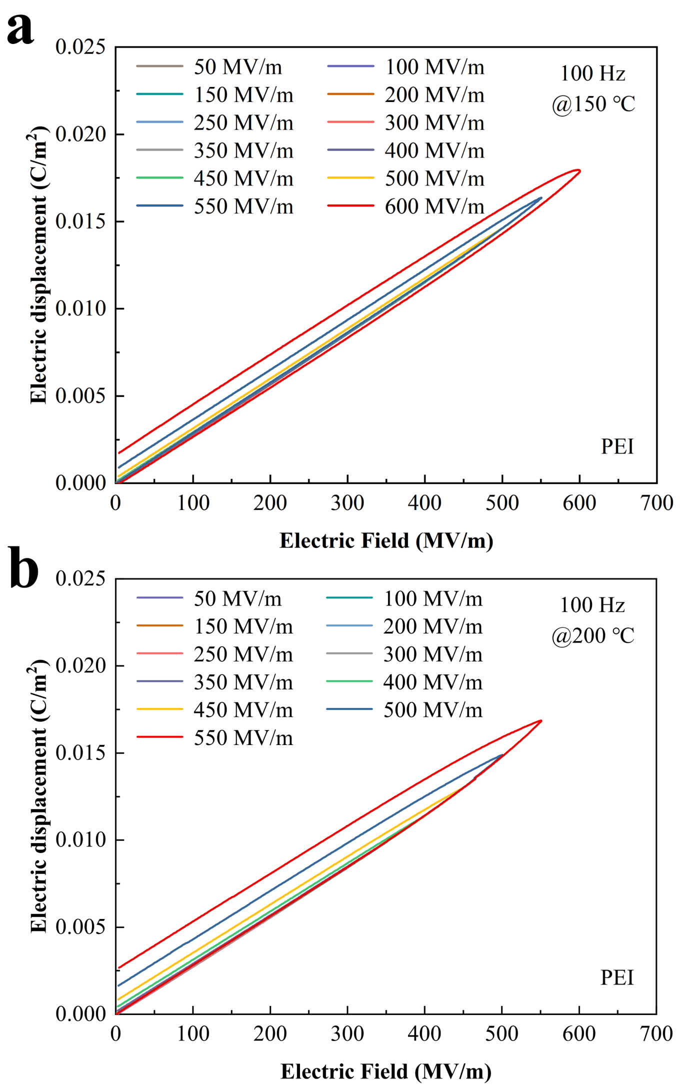

Supplementary Figure S37. Unipolar *D*-*E* loops of pristine PEI measured at (a) 150 °C and (b) 200 °C with 100 Hz.

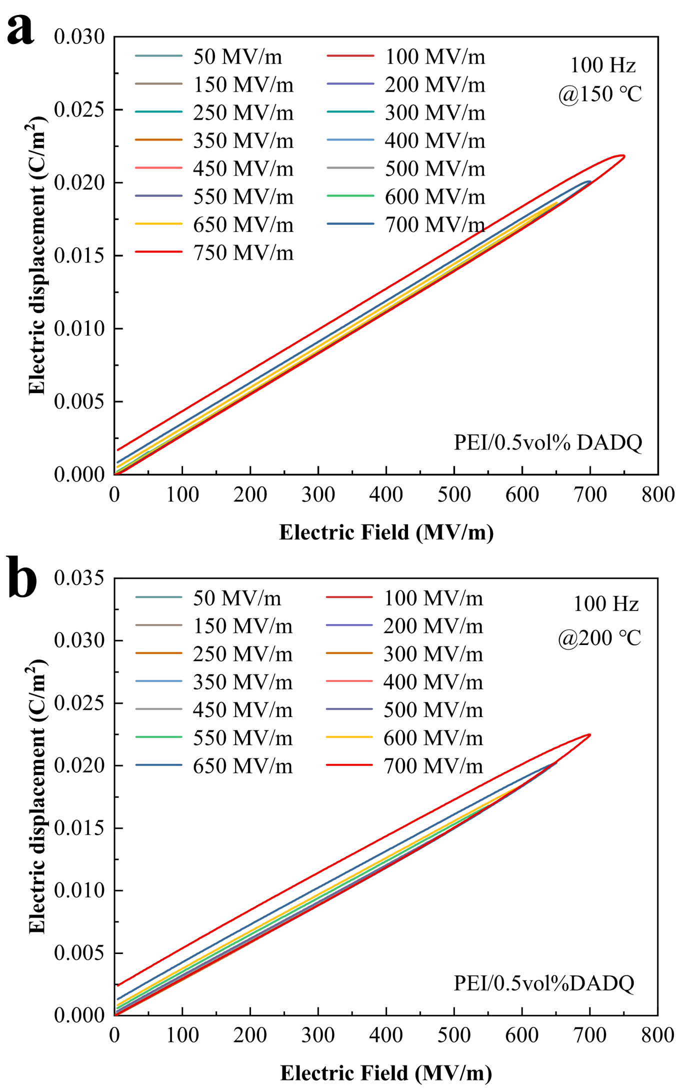

Supplementary Figure S38. Unipolar *D*-*E* loops of PEI/DADQ (0.5 vol%) measured at (a) 150 °C and (b) 200 °C with 100 Hz.

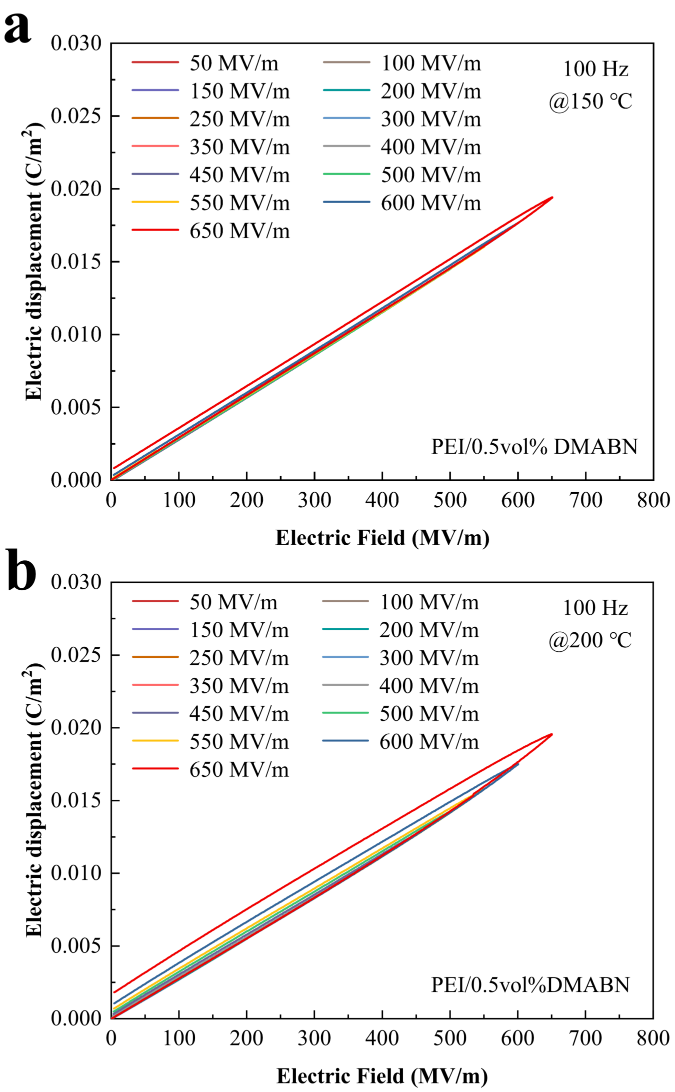

Supplementary Figure S39. Unipolar *D-E* loops of PEI/DMABN (0.5 vol%) measured at (a) 150 °C and (b) 200 °C with 100 Hz.

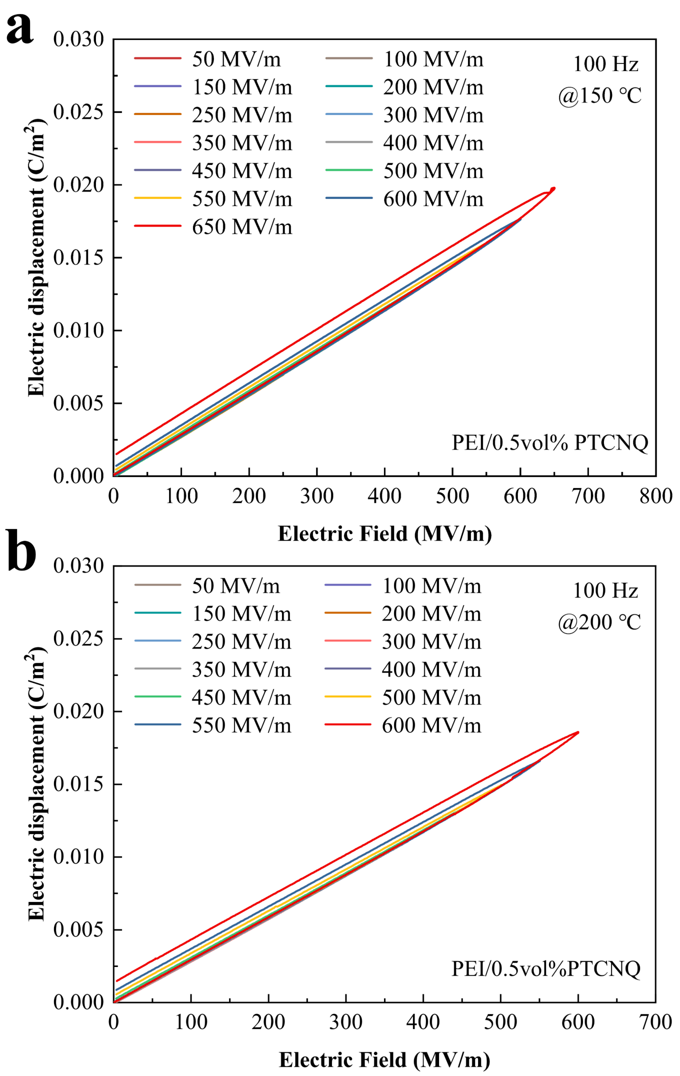

Supplementary Figure S40. Unipolar *D-E* loops of PEI/PTCNQ (0.5 vol%) measured at (a) 150 °C and (b) 200 °C with 100 Hz.

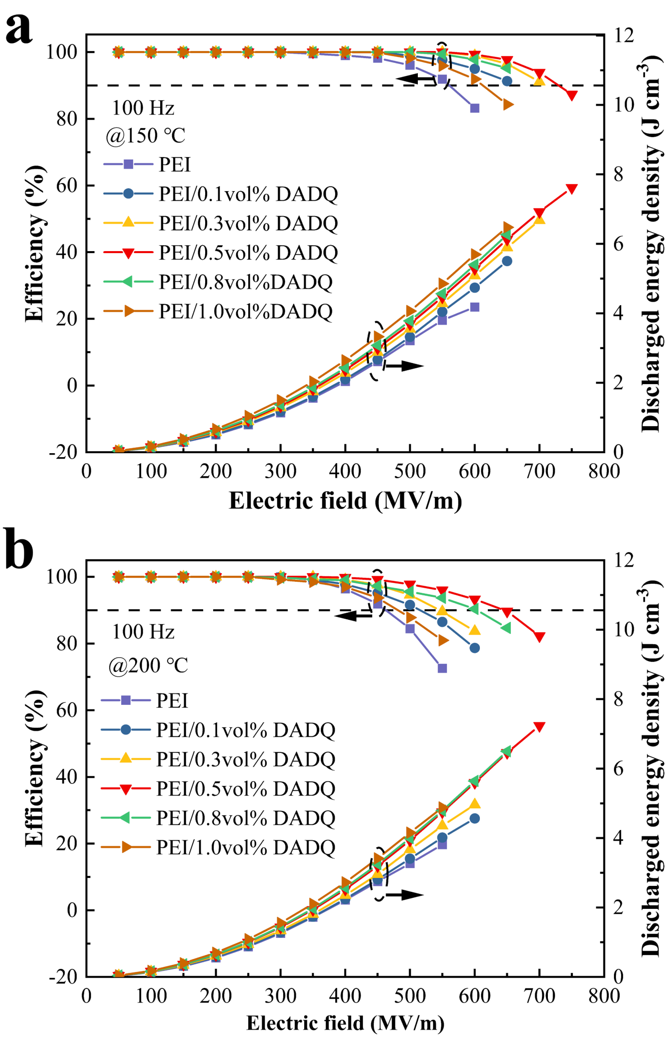

Supplementary Figure S41. Discharged energy density and efficiency of PEI/DADQ with various DADQ contents measured at (a) 150 °C and (b) 200 °C with 100 Hz.

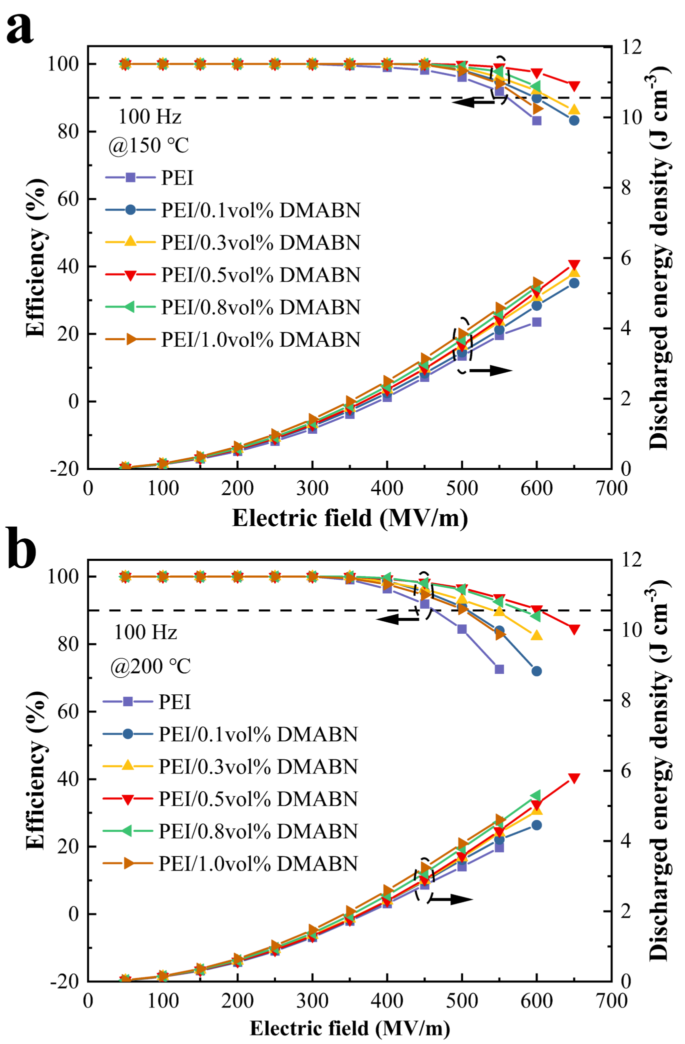

Supplementary Figure S42. Discharged energy density and efficiency of PEI/DMABN with various DMABN contents measured at (a) 150 °C and (b) 200 °C with 100 Hz.

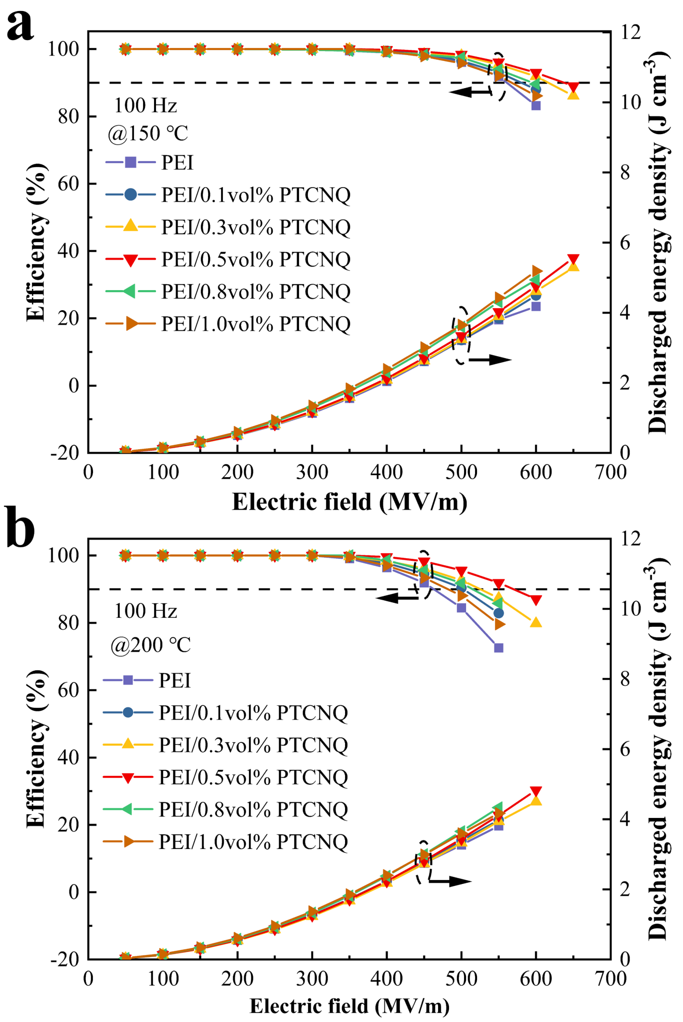

Supplementary Figure S43. Discharged energy density and efficiency of PEI/PTCNQ with various PTCNQ contents measured at (a) 150 ℃ and (b) 200 ℃ with 100 Hz.

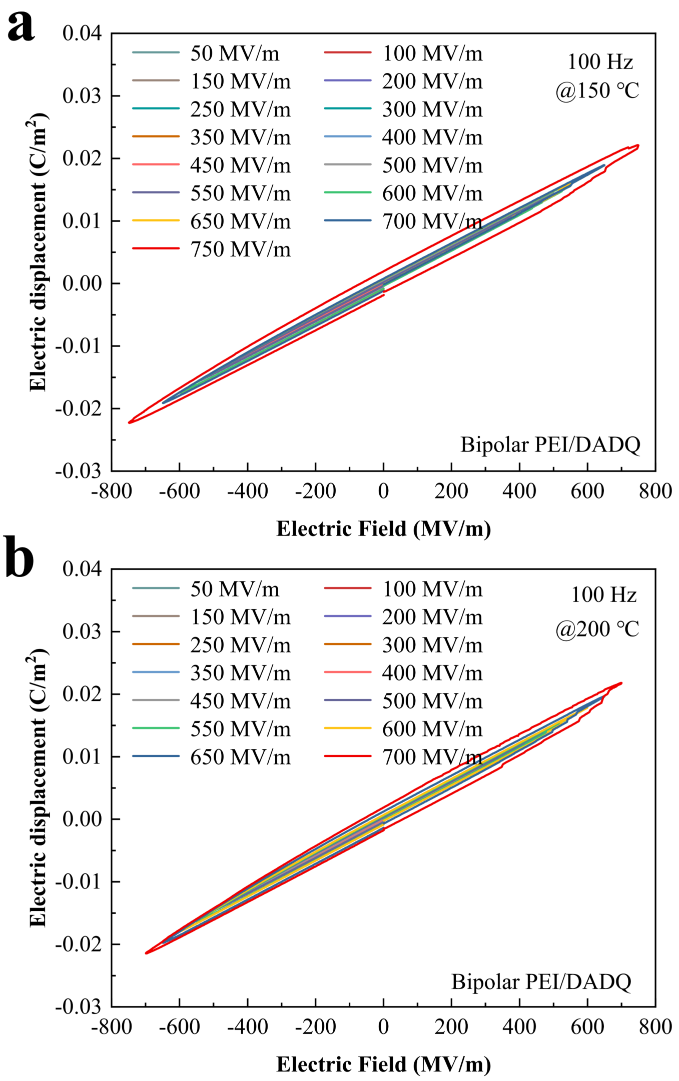

Supplementary Figure S44. Bipolar *D*-*E* loops of PEI/DADQ (0.5 vol%) measured at (a) 150 °C and (b) 200 °C with 100 Hz.

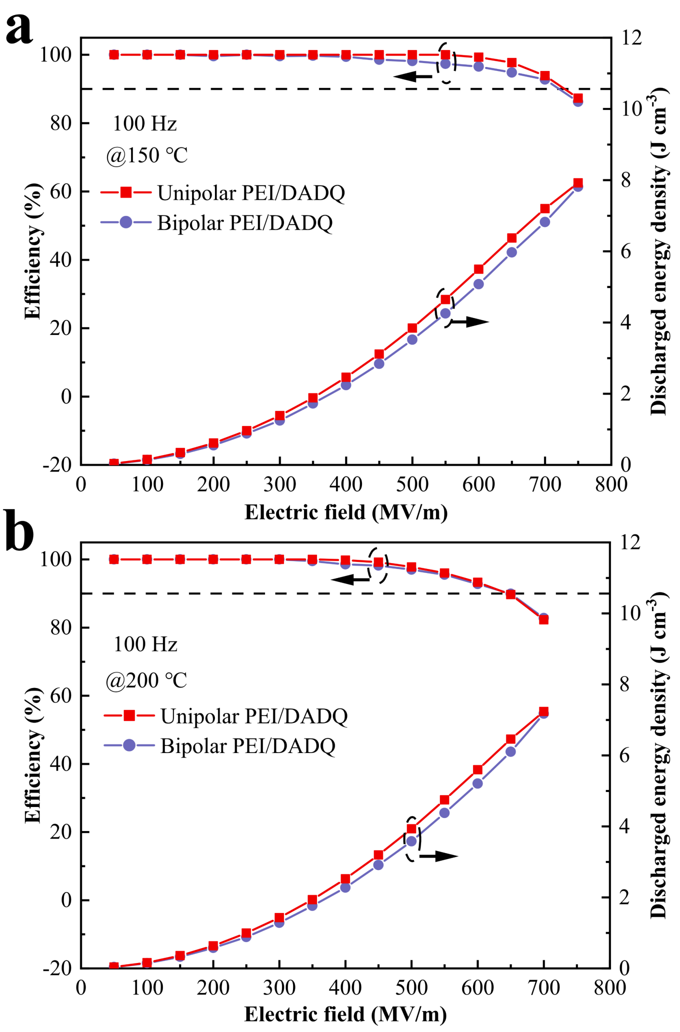

Supplementary Figure S45. Comparison of efficiency and discharged energy density of PEI/DADQ derived from unipolar and bipolar loops at (a) 150 ℃ and (b) 200 ℃ with 100 Hz.

Supplementary Table S4. Energy storage performance comparison among organic/inorganic hybrid dielectric films at 150 °C

Dielectric materials	Frequency (Hz)	Electric field at η>90% (MV/m)	Discharged energy density (J cm^{-3})	Ref.
PI/Al_2O_3	10	300	1.51	*Adv. Energy Mater.* **10**, 1903881 (2020).[1]
Al_2O_3-BT@SiO_2/PI-Al_2O_3	100	350	2.4	*Nano Energy* **99**, 107314 (2022).[2]
c-BCB/BNNs	10	400	2.2	*Nature* **523**, 576-579 (2015).[3]
XL-VK-2	10	400	2.76	*Energy Environ. Sci.* **13**, 1279-1286 (2020).[4]
c-BCB/Al_2O_3 NPLs	10	450	3.3	*Adv. Mater.* **31**, 1900875 (2019).[5]
A-P-A-P-A	100	400	4.35	*Adv. Funct. Mater.* **31**, 2102644 (2021).[6]
PEI/MOFs	100	500	3.5	*Mater. Horiz.* **10**, 3651-3659 (2023).[7]
PEEU/Al_2O_3	10	500	5	*Sci. Adv.* **6**, eaax6622 (2020).[8]
PEI/PCBM	10	550	4.5	*Nat. Commun.* **11**, 3919 (2020).[9]
o-POFNB	100	600	4.8	*Energy Environ. Sci.* **15**, 1307-1314 (2022).[10]
S-15-28	100	500	6.3	*J. Mater. Chem. A* **11**, 20021-20030 (2023).[11]
PSBNP-co-PTNI$_{0.02}$	10	600	6.18	*Nature* **615**, 62-66 (2023).[12]
PI-3.0	100	610	5.25	*Adv. Mater.* **35**,

				2211487 (2023).[13]
PEI/NTCDA	100	630	5.2	*Adv. Funct. Mater.* **33**, 2210050 (2022).[14]
F-PI/PCBM	100	709	6.39	*Adv. Mater.* **34**, 2207421 (2022).[15]
CS-ODA	100	650	7.02	*Adv. Mater.* **35**, 2207580 (2023).[16]

Supplementary Table S5. Energy storage performance comparison among organic/inorganic hybrid dielectric films at 200 °C

**Dielectric materials**	**Frequency (Hz)**	**Electric field at *η*>90% (MV/m)**	**Discharged energy density (J cm^{-3})**	**Ref.**
SO_2-PIM	10	200	1	*Mater. Horiz.* **7**, 592-597 (2020).[17]
PTFE	100	350	1.08	*Appl. Mater. Today* **21**, 100882 (2020).[18]
c-BCB/Al_2O_3 NPLs	10	300	1.4	*Adv. Mater.* **31**, 1900875 (2019).[5]
A-P-A-P-A	100	300	1.59	*Adv. Funct. Mater.* **31**, 2102644 (2021).[6]
Al_2O_3-BT@SiO_2/PI-Al_2O_3	100	300	1.8	*Nano Energy* **99**, 107314 (2022).[2]
PEIs/PI/PEIs	100	300	2	*Nano Energy* **97**, 107215 (2022).[19]
o-POFNB	100	400	2.2	*Energy Environ. Sci.* **15**, 1307-1314 (2022).[10]
ht-PEKNA	100	270	2.6	*Macromol. Mater. Eng.* **305**, 1900820 (2020).[20]
PEI/NTCDA	100	450	2.6	*Adv. Funct. Mater.* **33**, 2210050 (2022).[14]
CBDA/DCHM	100	400	2.81	*Mater. Horiz.* **10**, 2139-2148 (2023).[21]
PEI/PCBM	10	450	3	*Nat. Commun.* **11**, 3919 (2020).[9]
PI-3.0	100	525	3.45	*Adv. Mater.* **35**,

				2211487 (2023).[13]
F-PI/PCBM	100	572	4.39	*Adv. Mater.* **34**, 2207421 (2022).[15]
FPE/ICIT-Cl	100	560	4.8	*Adv. Energy Mater.* **13**, 2203961 (2023).[22]
PI-oxo	100	600	5.2	*Nat. Commun.* **14**, 2406, (2023).[23]
PSBNP-co-$PTNI_{0.02}$	10	550	5.34	*Nature* **615**, 62-66 (2023).[12]
Al-2 PI	100	639	5.74	*Energy Storage Mater.* **65**, 103095 (2024).[24]
FPI@DG	100	675	6.21	*Nat. Commun.* **15**, 8647 (2024).[25]

Supplementary References

1. Ai, D. et al. Tuning nanofillers in in situ prepared polyimide nanocomposites for high-temperature capacitive energy storage. *Adv. Energy Mater.* **10**, 1903881 (2020).
2. Dong, J. et al. Enhancing high-temperature capacitor performance of polymer nanocomposites by adjusting the energy level structure in the micro-/meso-scopic interface region. *Nano Energy* **99**, 107314 (2022).
3. Li, Q. et al. Flexible high-temperature dielectric materials from polymer nanocomposites. *Nature* **523**, 576-579 (2015).
4. Li, H. et al. Crosslinked fluoropolymers exhibiting superior high-temperature energy density and charge-discharge efficiency. *Energy Environ. Sci.* **13**, 1279-1286 (2020).
5. Li, H. et al. Scalable polymer nanocomposites with record high-temperature capacitive performance enabled by rationally designed nanostructured inorganic fillers. *Adv. Mater.* **31**, 1900875 (2019).
6. Dong, J. et al. A facile in situ surface-functionalization approach to scalable laminated high-temperature polymer dielectrics with ultrahigh capacitive performance. *Adv. Funct. Mater.* **31**, 2102644 (2021).
7. Li J. et al. Thermally activated dynamic bonding network for enhancing high-temperature energy storage performance of PEI-based dielectrics. *Mater. Horiz.* **10**, 3651-3659 (2023).
8. Zhang T. et al. A highly scalable dielectric metamaterial with superior capacitor performance over a broad temperature. *Sci. adv.* **6**, eaax6622 (2020).
9. Yuan, C. et al. Polymer/molecular semiconductor all-organic composites for high-temperature dielectric energy storage. *Nat. Commun.* **11**, 3919 (2020).
10. Deshmukh, A. A. et al. Flexible polyolefin dielectric by strategic design of organic modules for harsh condition electrification. *Energy Environ. Sci.* **15**, 1307-1314 (2022).
11. Duan Y. et al. High-temperature resistant polyetherimides containing a twisted spirane structure for capacitive energy storage. *J. Mater. Chem. A* **11**, 20021-20030 (2023).
12. Chen, J. et al. Ladderphane copolymers for high-temperature capacitive energy storage. *Nature* **615**, 62-66 (2023).
13. Dong, J. et al. Scalable Polyimide-organosilicate hybrid films for high-temperature capacitive energy storage. *Adv. Mater.* **35**, 2211487 (2023).
14. Zhang, B. et al. Superior high-temperature energy density in molecular semiconductor/polymer all-organic composites. *Adv. Funct. Mater.* **33**, 2210050 (2022).
15. Ren, W. et al. Scalable ultrathin all-organic polymer dielectric films for high-temperature capacitive energy storage. *Adv. Mater.* **34**, 2207421 (2022).
16. Pan, Z. et al. Tailoring poly(styrene-co-maleic anhydride) networks for all-polymer dielectrics exhibiting ultrahigh energy density and charge-discharge efficiency at elevated temperatures. *Adv. Mater.* **35**, 2207580 (2023).
17. Zhang Z. et al. High-κ polymers of intrinsic microporosity: a new class of high

temperature and low loss dielectrics for printed electronics. *Mater. Horiz.* **7**, 592-597 (2020).

18. Luo S. et al. Elaborately fabricated polytetrafluoroethylene film exhibiting superior high-temperature energy storage performance. *Appl. Mater. Today* **21**, 100882 (2020).
19. Niu, Y. et al. Significantly enhancing the discharge efficiency of sandwich-structured polymer dielectrics at elevated temperature by building carrier blocking interface. *Nano Energy* **97**, 107215 (2022).
20. Xu D. et al. Rational design of soluble polyaramid for high-efficiency energy storage dielectric materials at elevated temperatures. *Macromol. Mater. Eng.* **305**, 1900820 (2020).
21. Song J. et al. Alicyclic polyimides with large band gaps exhibit superior high-temperature capacitive energy storage. *Mater. Horiz.* **10**, 2139-2148 (2023).
22. Zhou, Y., Zhu, Y., Xu, W. & Wang, Q. Molecular trap engineering enables superior high-temperature capacitive energy storage performance in all-organic composite at 200 °C. *Adv. Energy Mater.* **13**, 2203961 (2023).
23. Wang, R. et al. Designing tailored combinations of structural units in polymer dielectrics for high-temperature capacitive energy storage. *Nat. Commun.* **14**, 2406, (2023).
24. Ren W. et al. Metallized stacked polymer film capacitors for high-temperature capacitive energy storage. *Energy Storage Mater.* **65**, 103095 (2024).
25. Yang M. et al. Enhanced high-temperature energy storage performances in polymer dielectrics by synergistically optimizing band-gap and polarization of dipolar glass. *Nat. Commun.* **15**, 8647 (2024).
