# Supplementary material for: Enhancing energetic disorder in all-organic composite dielectrics for high-temperature capacitive energy storage
Source: Nat Commun. 2025 Jul 1;16:5620. doi: 10.1038/s41467-025-60741-1 (PMC12216148; doi:10.1038/s41467-025-60741-1)
Supplement: Supplementary file 2 — Description of Additional Supplementary Files [file 41467_2025_60741_MOESM2_ESM.pdf]

Description of Additional Supplementary Files

File Name: Supplementary Data file

Description: Computational modelling data of distribution of electronic energy levels, Interaction energy fluctuations and Polymer configurations.
